# Sex-specific dominance reversal of genetic variation for fitness

**DOI:** 10.1371/journal.pbio.2006810

**Published:** 2018-12-11

**Authors:** Karl Grieshop, Göran Arnqvist

**Affiliations:** Department of Ecology and Genetics, Animal Ecology, Uppsala University, Uppsala, Sweden; The Institute of Science and Technology Austria, Austria

## Abstract

The maintenance of genetic variance in fitness represents one of the most longstanding enigmas in evolutionary biology. Sexually antagonistic (SA) selection may contribute substantially to maintaining genetic variance in fitness by maintaining alternative alleles with opposite fitness effects in the two sexes. This is especially likely if such SA loci exhibit sex-specific dominance reversal (SSDR)—wherein the allele that benefits a given sex is also dominant in that sex—which would generate balancing selection and maintain stable SA polymorphisms for fitness. However, direct empirical tests of SSDR for fitness are currently lacking. Here, we performed a full diallel cross among isogenic strains derived from a natural population of the seed beetle *Callosobruchus maculatus* that is known to exhibit SA genetic variance in fitness. We measured sex-specific competitive lifetime reproductive success (i.e., fitness) in >500 sex-by-genotype F_1_ combinations and found that segregating genetic variation in fitness exhibited pronounced contributions from dominance variance and sex-specific dominance variance. A closer inspection of the nature of dominance variance revealed that the fixed allelic variation captured within each strain tended to be dominant in one sex but recessive in the other, revealing genome-wide SSDR for SA polymorphisms underlying fitness. Our findings suggest that SA balancing selection could play an underappreciated role in maintaining fitness variance in natural populations.

## Author summary

Evolution requires genetic variation, but selection will tend to fix whichever alleles confer the highest fitness, depleting the genetic variation upon which it acts. Sexually antagonistic (SA) genetic variation—in which alternative alleles have opposite fitness effects in the sexes—can generate balancing selection that maintains genetic variation for fitness if the alleles that benefit a given sex are also dominant in that sex. Here, we show that the SA genetic variation underlying fitness in a well-known seed beetle population exhibits these beneficial reversals of dominance, suggesting SA selection may commonly maintain heritable genetic variation for fitness throughout the genome.

## Introduction

One of the most longstanding challenges for evolutionary biologists has been to explain the maintenance of genetic variance in fitness [1–7]. Selection should erode genetic variation as it eliminates deleterious alleles and fixes beneficial ones. Yet natural populations harbor abundant heritable variation for fitness and life history traits [8–9]. The two general hypotheses for explaining this are mutation-selection balance and balancing selection [2–4]. Under the former, many polymorphisms throughout the genome are maintained at low allele frequencies because of a constant influx of deleterious mutations [10–14], yet this process cannot single-handedly explain the extent and pattern of the genetic variance observed in nature [4–7] (explained below). Thus, some form of balancing selection—including scenarios in which alternative alleles offer fitness benefits in different contexts (e.g., environments, genotypes, seasons, or sexes)—must contribute to the maintenance of polymorphisms for fitness throughout the genome [4–7].

**Fig 1:**
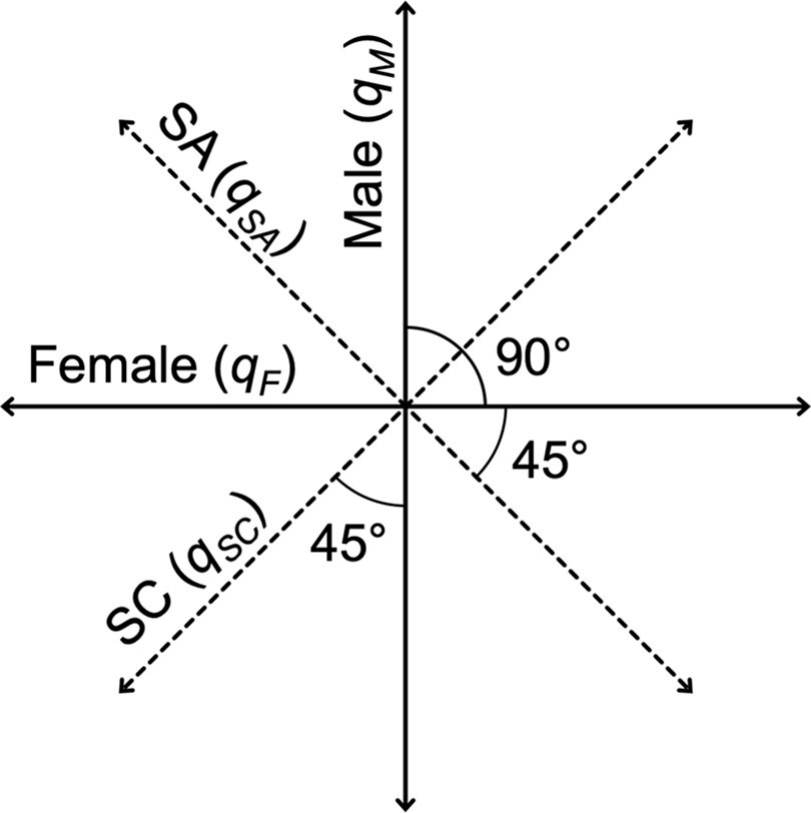
Geometric definition of terms and concepts. For a given inheritance class *Q* (e.g., additivity, dominance, epistasis, etc.; see below), the BLUPs for each strain’s male- (*q*_*M*_) and female-specific (*q*_*F*_) variance components from models that were fit separately to male and female data sets represent axes of variation that can be plotted in a bivariate relationship (solid lines). These coordinate systems can be rotated 45° (see Materials and methods) to derive the SA (*q*_*SA*_) and SC (*q*_*SC*_) dimensions (dashed lines) for each inheritance class. BLUP, best linear unbiased prediction; SA, sexually antagonistic; SC, sexually concordant.

Sexually antagonistic (SA) selection can cause alternative alleles to have opposite fitness effects in males and females [15–18] and has the potential to be the most widespread source of balancing selection among eukaryotes. Sex is a nearly ubiquitous feature of eukaryotic life [19], SA genetic variation is an inevitable outcome of two sexes sharing the same genome whilst having different fitness optima [20–22], and antagonistic forms of balancing selection should generate more stable (less-transient) polymorphisms for fitness than nonantagonistic forms of balancing selection [23]. Further, well-adapted populations should exhibit an overabundance of SA genetic variance in fitness relative to sexually concordant (SC) genetic variance (i.e., that which affects the sexes similarly; see Fig 1) because (1) purifying selection should remove SC genetic variation relatively efficiently [24], and (2) the rate at which SA polymorphisms can be resolved should be low relative to the rate at which novel SA mutations occur [15,17–18,25–29]. A growing body of evidence for standing SA genetic variation in natural and laboratory populations largely supports these predictions (e.g., [26,30–40]).

The capacity for SA selection to generate balancing selection that results in stable polymorphisms for fitness drastically increases with sex-specific dominance reversal (SSDR) [41–42], in which the alleles that benefit a given sex’s fitness are also dominant in that sex, generating a net heterozygote advantage in the population. Such beneficial reversals of dominance, in the more general case of antagonistic pleiotropy [43], were met with early skepticism [44–46], but more recent theory is changing that view. The average locus underlying two polygenic homologous phenotypes with antagonistic fitness effects (e.g., male and female fitness) is actually expected to exhibit at least partial dominance reversal for fitness under the nonrestrictive assumption that the fitness functions are concave in the vicinity of their overlap [47]. Further, SA genetic variation should in turn select for modifier loci that enable heterozygous males and females to exhibit favorable dominance relationships between SA alleles [48], making SSDR of SA genetic variation for fitness a predicted outcome of adaptive evolution.

The ability of SSDR to promote the maintenance of SA polymorphisms for fitness has been known for decades [41], yet there are currently no direct empirical tests of SSDR for fitness. Barson and colleagues [39] recently demonstrated SSDR for a single major-effect locus affecting age at maturity in salmon. Fitness, however, is a highly polygenic trait, and investigating the properties of many loci typically requires a quantitative genetic approach [49–50].

The full diallel cross [50] is the premier quantitative genetic breeding design, capable of partitioning phenotypic variance into that attributable to additive genetic effects, parental effects, dominance, and epistasis. This enables tests of several of the hypotheses for the maintenance of genetic variance in fitness. Because the many weakly deleterious alleles maintained by mutation-selection balance should necessarily exhibit partial dominance [4,7,10–14,51–52], it should generate pronounced additive genetic variance relative to dominance variance if it is the predominant contributor to fitness variance. By contrast, balancing selection acting to maintain relatively fewer polymorphisms of larger effect size, which may involve dominance coefficients up to and including complete dominance, would generate pronounced dominance variance relative to additive genetic variance if it predominates [4,7–8,50]. As for distinguishing among the many forms of balancing selection, diallel data offer the possibility to distinguish SA balancing selection from other forms of balancing selection by testing for its hallmark SSDR (see above). To this end, variance partitioning is not a sufficient test. Rather, full diallel data offer the possibility to quantify the relative amount of fixed recessive allelic variation among a set of inbred strains, and if the data are sex-specific, then it enables one to do this for males and females independently. A positive correlation among strains for their relative amounts of recessive allelic variation when measured in males versus females would demonstrate that strains tend to exhibit a relatively high or low number of fixed recessive alleles (regardless of which sex they are expressed in), whereas a negative correlation would demonstrate that strains tend to be fixed for allelic variation that is recessive in one sex but dominant in the other (i.e., SSDR). It is conceivable that SSDRs of this nature could evolve in the context of SC allelic variants that merely exhibit sex differences in the relative strength of selection acting on each of them. Thus, the most conservative and explicit test of SSDR for the SA genetic variation underlying fitness would be to analyze this correlation after statistically removing SC additive genetic effects from the data.

**Fig 2:**
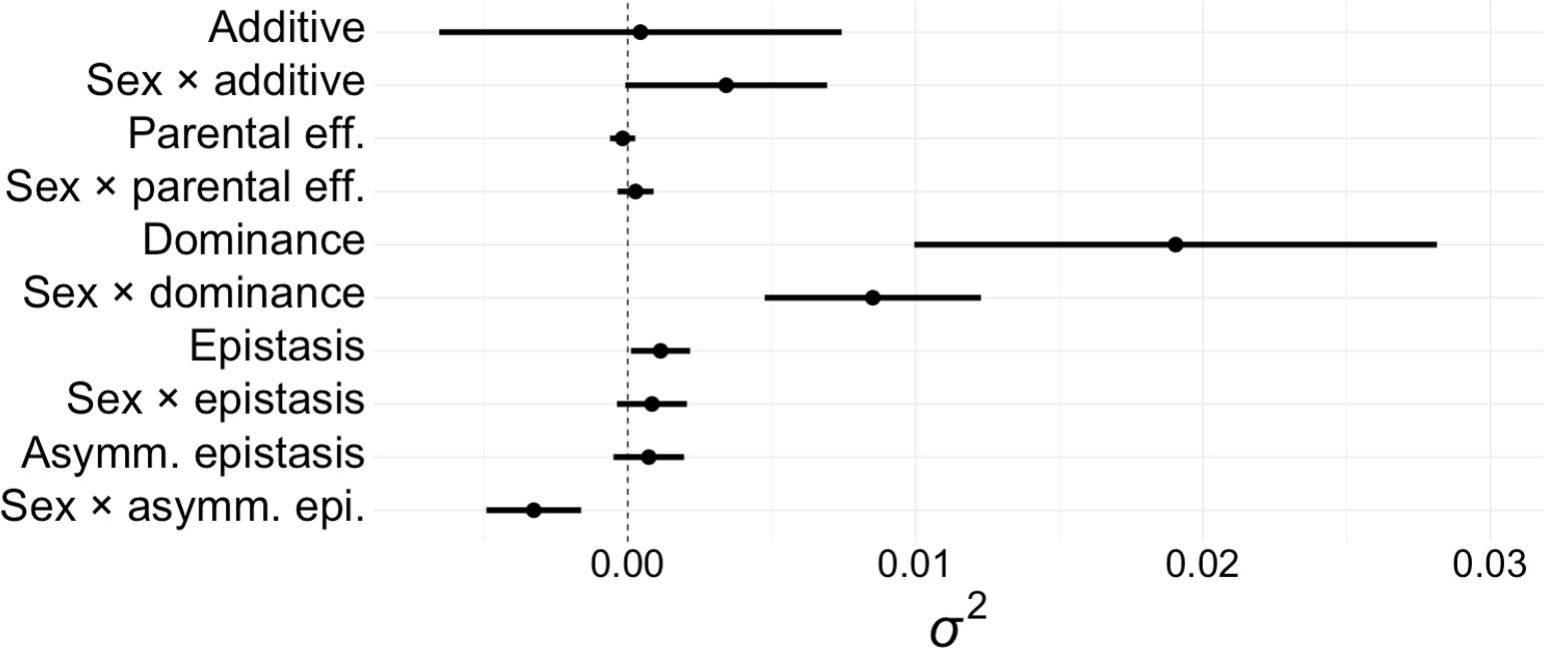
Graphical representation of variance partitioning. Variance components (*σ*^2^, ± 1 s.e.) for fitness estimated by REML. The data underlying all figures and tables can be found in S1 Data. asymm., asymmetric; eff., effects; epi., epistasis; REML, restricted maximum likelihood; s.e., standard error.

**Fig 3:**
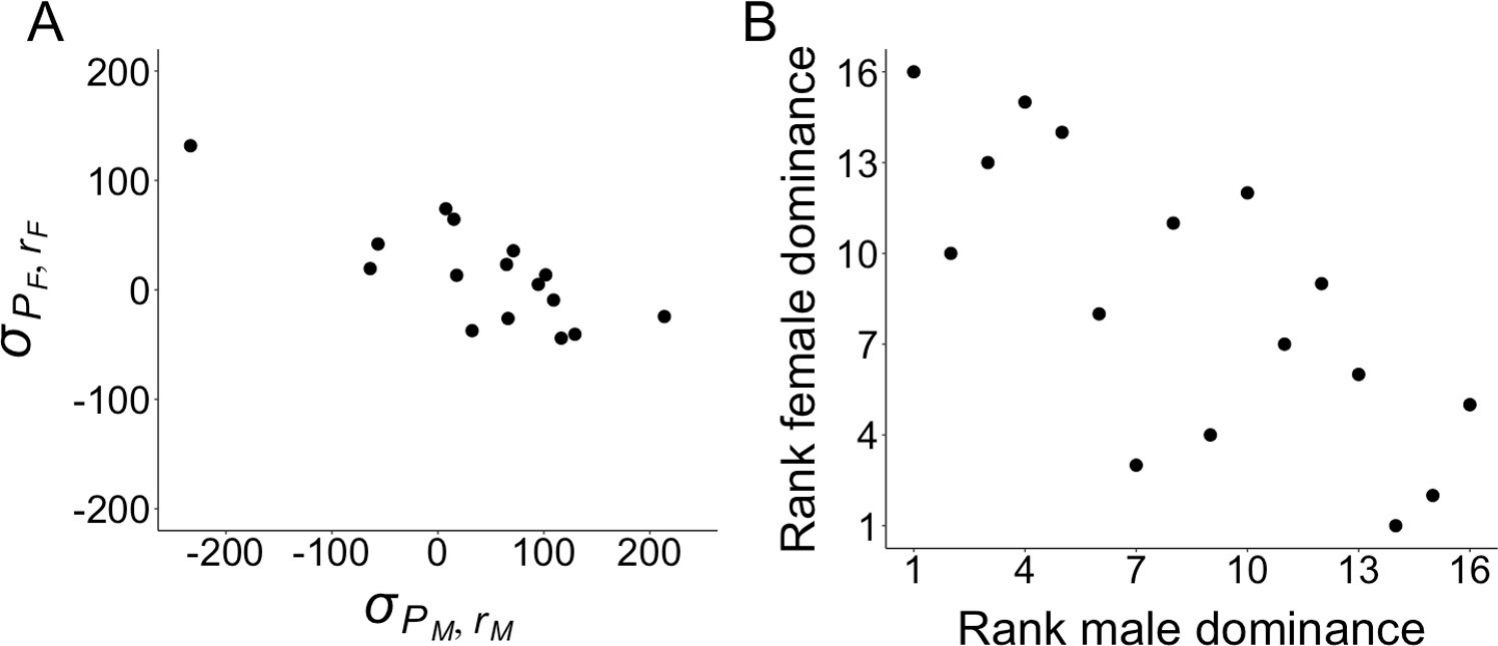
SSDR of the allelic variation underlying fitness. (A) The relative amount of recessive allelic variation for fitness in males ($\sigma_{{P_{M},r}_{M}}$) and females ($\sigma_{{P_{F},r}_{F}}$) was significantly negatively correlated ($r_{\sigma_{{P_{M},r}_{M}},\sigma_{{P_{F},r}_{F}}}$ = −0.779 [95% CI −0.92 to −0.46], *P* = 0.0004) across strains (*N* = 16; units reflect nonstandardized residual fitness from a model that accounted for environmental and epistatic variance), and (B) the same relationship illustrated and analyzed as ranks (i.e., strains ranked in order of their relative “dominance” over one another; $r_{\sigma_{{P_{M},r}_{M}},\sigma_{{P_{F},r}_{F}}}$: −0.738, *P* = 0.0016). Strains tended to be enriched with allelic variation for fitness that was dominant in their heterozygous sons but recessive in their heterozygous daughters and vice versa. SSDR, sex-specific dominance reversal.

Here, we used a full diallel cross among 16 isogenic strains to partition genetic variance in sex-specific competitive lifetime reproductive success (hereafter “fitness”) in a wild-caught population of the seed beetle *C*. *maculatus* that is known to exhibit pronounced SA genetic variance in fitness [37–38,53]. This species exhibits a polyandrous mating system, X/Y sex determination, and pronounced sexual dimorphism and sex-biased gene expression [33,38,54–56]. In total, 3,278 individual fitness assays (1,731 male and 1,547 female) were conducted over the 256 possible cross types (hereafter “families”)—i.e., the 240 outcrossed (heterozygous) families and 16 parental-self (homozygous) families of a full 16 × 16 diallel. Considering that these inbred strains originate from a population whose genetic variance in fitness is predominantly SA [37], the present findings of pronounced dominance variance and sex-specific dominance variance relative to additive genetic variance (Fig 2) suggest that this population’s fitness variance is largely underlain by relatively few, large-effect polymorphisms under SA selection, as opposed to many small-effect polymorphisms in mutation-selection balance [4,7–8,50]. Our analyses revealed that strains exhibited significantly negative rank correlation for their relative amount of fixed dominant alleles for fitness when measured in males versus females (Fig 3). Thus, whether the average allele underlying fitness in this population is dominant or recessive in a heterozygote depends on whether it is being expressed in a male or a female. As mentioned above, this could still be the case for some SC allelic variation as well, but this relationship remained strong when the SC additive genetic effects were statistically removed from the data beforehand (S1 Fig), explicitly demonstrating SSDR for the SA genetic variation underlying fitness. Our findings are consistent with genome-wide SSDR maintaining balanced SA polymorphisms for fitness, which has important implications for the capacity of SA selection to explain fitness variance in natural populations.

## Results

We performed a full diallel cross among 16 isogenic strains, generating an F_1_ generation of 240 possible outcrossed families and 16 parental selfs. We assayed F_1_ male and female competitive lifetime reproductive success (i.e., fitness) separately to obtain sex-specific measures of fitness for each outcrossed family and selfed strain (S2 Fig). Phenotypic variance in fitness was partitioned into that attributable to the overall fixed effects of replicate block (*x*), inbreeding (*b*_1_), sex (*S*), and sex-specific inbreeding (*S*×*b*_1_), as well as the following strain- and cross-specific random effects (i.e., variance components) using restricted maximum likelihood (REML) estimation: additive genetic variance (*a*), dominance (*b*_2_), parental effects (*c*), symmetric epistasis (*b*_3_), asymmetric epistasis (*d*), and sex-specific versions thereof (i.e., their interaction with *S*). For this REML approach, we used the FDIALLEL procedure [57] for GenStat (v.18.2.0.18409; [58]) to fit a custom version of Hayman’s [59] model that we modified to accommodate sex-specific effects (hereafter the “full sexed” model). We also applied Lenarcic and colleagues’ [60] full sexed hierarchical Bayesian model fit to our data using the “BayesDiallel” MCMC Gibbs sampler [61] for R (v.3.2.1; [62]). The two approaches yielded qualitatively similar results (the Bayesian approach is reported in the S3–S5 Figs, S1 and S2 Tables, and S1 Text).

To aid interpretation, separate male and female models were also performed (on separate male and female data sets), which provided the sex-specific variance components (*q*_*M*_ and *q*_*F*_ of Fig 1) that were used in the following geometric interpretation of “sexed” and “unsexed” variance components from the full sexed model (see below) for all inheritance classes (see S3 Table for separate sex-specific variance component estimates). For a given inheritance class *Q* (e.g., additivity, dominance, epistasis, etc.), the full sexed model provides sexed (*S*×*q*) and unsexed (*q*) variance components. Strains’ best linear unbiased predictions (BLUPs) for a given variance component represent their estimated values along those axes of variation. All unsexed axes of variation (*q*) from the full sexed model were highly correlated to (i.e., ≈) the derived SC dimension of their respective inheritance class (*q*_*SC*_; all Pearson’s correlation coefficients $r_{q,q_{SC}}$ were > 0.98, all *P* values < 0.0001; see Materials and methods and Fig 1). Thus, all unsexed variance components from the full sexed model were found to represent SC effects. This was true even in cases of technically improper correlations in which the standard error of *q*, *q*_*M*_, or *q*_*F*_ overlapped zero (e.g., $r_{a,a_{SC}}$ = 0.99, *P* < 0.0001, despite *a* being approximately 0; see below). Further, the sexed variance components for additivity (*S*×*a*), dominance (*S*×*b*_2_), and epistasis (*S*×*b*_3_) were found to represent SA effects: the inner product between these unsexed (i.e., SC, see above) and sexed effects revealed them as describing approximately orthogonal axes of variation (rendering the latter SA; see Fig 1). That is, their BLUPs fell along axes of variation that were at approximately 90° angular displacements, *θ*, from one another (*θ*_*a*,*S*×*a*_ = 91.50°, $\theta_{b_{2},S\times b_{2}}$ = 90.00°, $\theta_{b_{3},S\times b_{3}}$ = 91.49°). Negative variance component estimates for parental effects (*c*) and sex-specific asymmetric epistasis (*S*×*d*) rendered their BLUPs invalid and excluded their inheritance classes from this geometric interpretation. Again, all unsexed variance components describe SC effects, whereas the sex-specific additive (*S*×*a*), dominance (*S*×*b*_2_), and epistatic (*S*×*b*_3_) variance components (being orthogonal to their SC counterparts) describe SA effects (see Fig 1). We therefore refer to them accordingly (e.g., SA additive genetic variance). Further information on the terminology and meaning of variance components is available in the S1 Text.

### Variance partitioning

There were no overall differences between the sexes (*S*) in mean fitness (Table 1). The overall effect of inbreeding (*b*_1_) was large and statistically significant (Table 1). The sexes differed in this regard, as revealed by a significant sex-specific inbreeding effect (*S*×*b*_1_; Table 1). Separate male and female models revealed that the effect of inbreeding was stronger in males than females (S3 Table), a result confirmed by other studies (e.g., [63]). Note that these sizeable inbreeding and sex-specific inbreeding effects have been accounted for as overall (fixed) effects prior to the estimation of random effects variance components and therefore do not inflate or disrupt estimates of dominance (*b*_2_), sex-specific dominance (*S*×*b*_2_), or any other variance component.

**Table 1:** Results of the full sexed REML model. F statistics with *P* values for the overall (fixed) effects and variances (*σ*^2^) with s.e. for strain- and cross-specific (random) effects (i.e., variance components).

	Effect	Symbol	F	*P*
Overall	Sex	*S*	3.82	0.069
	Inbreeding	*b*_1_	28.69	<0.001
	Sex × inbreeding	*Sb*_1_	7.74	0.015
	Block	*x*	87.72	<0.001
			***σ*^2^**	**s.e.**
Strain- and cross-specific	Additive	*a*	0.0004	0.0070
	Sex × additive	*S*×*a*	0.0034	0.0035
	Parental eff.	*c*	−0.0002	0.0004
	Sex × parental eff.	*S*×*c*	0.0003	0.0006
	Dominance	*b*_2_	0.0191	0.0091
	Sex × dominance	*S*×*b*_2_	0.0085	0.0038
	Epistasis	*b*_3_	0.0011	0.0010
	Sex × epistasis	*S*×*b*_3_	0.0008	0.0012
	Asymm. epistasis	*d*	0.0007	0.0012
	Sex × asymm. epi.	*S*×*d*	−0.0033	0.0017
	Error	*ε*	0.0963	0.0026

Abbreviations: asymm., asymmetric; eff., effects; epi., epistasis; REML, restricted maximum likelihood; s.e., standard error.

In general, inheritance for fitness was characterized by pronounced SC dominance (*b*_2_) and SA dominance (*S*×*b*_2_; Table 1 and Fig 2). There was also a relatively small but nonzero contribution from SC epistatic variance (*b*_3_; Table 1 and Fig 2). Of those, *b*_2_ and *S*×*b*_2_, respectively, contributed approximately 17 times and approximately 8 times the genetic variance to fitness relative to *b*_3_ (Table 1).

Although the standard error of SA additive genetic variance (*S*×*a*) did barely overlap zero, we note that its estimate was 10 times that of SC additive genetic variance (*a*) (Table 1 and Fig 2). Parental effects (*c*), sex-specific parental effects (*S*×*c*), SA epistasis (*S*×*b*_3_), asymmetric epistasis (*d*), and sex-specific asymmetric epistasis (*S*×*d*) were all estimated as near zero with standard errors overlapping zero (Table 1 and Fig 2).

Separate male and female models revealed about 5 times the residual variance (*ε*) for males than for females, complicating precise quantitative comparison between these models (S3 Table). Qualitatively, however, nonzero variance was estimated for dominance (*b*_2_) and epistasis (*b*_3_) in both male and female models, and these were the only inheritance classes with nonzero variance in either model (S3 Table). In both models, *b*_2_ was estimated at about 13 times that of *b*_3_ (S3 Table).

### Assessment of SSDR

A full diallel cross among isogenic strains offers additional valuable insight regarding the dominance relationships between the fixed allelic variants among strains [50,59]. In particular, an estimate of the relative proportion of dominant:recessive alleles among the homozygous parental strains is given by the array covariances between strains’ outcrossed (heterozygous) family means and the means of the inbred (homozygous) parental selfs of the strains to which they were crossed (S6 Fig [50,59]). Strains whose outcrossed (heterozygous) family means are determined by (i.e., covary with) the inbred (homozygous) means of the strains to which they are crossed harbor alleles that are apparently recessive to those of other strains. By contrast, strains whose outcrossed (heterozygous) family means are instead independent of (i.e., do not covary with) the inbred (homozygous) means of the strains to which they are crossed harbor alleles that are apparently dominant to those of other strains. After removing environmental and epistatic variance from the data, *σ*_*P*,*r*_, each strain’s covariance between its outcrossed family means (*r*) and the means of the inbred parental selfs (*P*) of the strains to which they were crossed [50,59] was used as an estimate of the relative amount of recessive alleles fixed within each strain (see Materials and methods and S6 Fig). This was done for male ($\sigma_{{P_{M},r}_{M}}$) and female ($\sigma_{{P_{F},r}_{F}}$) fitness separately, and these estimates were significantly negatively correlated (Pearson’s $r_{\sigma_{{P_{M},r}_{M}},\sigma_{{P_{F},r}_{F}}}$ = −0.779 (95% CI −0.92 to −0.46), *P* = 0.0004; Fig 3A). This effect was not driven by any particular strain(s), as evidenced by a significantly negative nonparametric rank correlation, indicating strains’ relative “dominance” over one another (Spearman’s $r_{\sigma_{{P_{M},r}_{M}},\sigma_{{P_{F},r}_{F}}}$: −0.738, *P* = 0.0016; Fig 3B). These tests remained strongly significant after first having statistically removed SC additive genetic variance (Pearson’s $r_{\sigma_{{P_{M},r}_{M}}\prime ,\sigma_{{P_{F},r}_{F}}\prime }$ = −0.665 (95% CI −0.87 to −0.25), *P* = 0.005; Spearman’s $r_{\sigma_{{P_{M},r}_{M}}\prime ,\sigma_{{P_{F},r}_{F}}\prime }$: −0.635, *P* = 0.0098; S1 Fig), explicitly demonstrating SSDR of the SA genetic variation for fitness.

## Discussion

Progress in our general understanding of the maintenance of genetic variance in fitness requires the identification of broadly applicable mechanisms. Knowing that mutation-selection balance cannot explain everything [4–7], the question for many evolutionary biologists becomes: which form(s) of balancing selection account for the remaining genetic variance? There are many mechanisms of balancing selection with theoretical and empirical support that are capable of maintaining stable polymorphisms for fitness under certain conditions. However, SA selection is potentially one of the most widespread among eukaryotes—pending the prevalence of SSDR—offering a general solution to this classic evolutionary question. Unfortunately, SA selection often goes unmentioned in reviews on the topic (e.g., [5–6]), leaving it relatively poorly understood from a modern genomics perspective [64–65]. We will therefore briefly review the inevitability of SA genetic variation, for which the present findings and other recent studies substantially strengthen the case for its role in maintaining genetic variance in fitness.

Antagonistic mutations (showing any form of antagonistic pleiotropy) sweep to fixation more slowly than nonantagonistic mutations, meaning that they will generate a weaker genomic signature of selection but will actually have a greater, more sustained contribution to genetic variance in fitness [23]. In accordance, experimental evolution in microorganisms often reveals that adaptation in a given context or environment comes at the price of reduced fitness in other contexts (reviewed in [66]), indicating substantial standing genetic variation with antagonistic pleiotropic effects. Likewise, Qian and colleagues [67] demonstrated widespread antagonistic pleiotropy in hundreds of genes across the yeast genome. The prevalence of these genetic trade-offs is expected to increase with increasing organismal complexity such as specialized tissues, developmental stages, and sexes [67–69]. Further, the resolution of such genetic trade-offs evolves more slowly under smaller effective population sizes and with longer generation times [67], again implying an even greater likelihood for antagonistic pleiotropy to maintain genetic variance in fitness in complex multicellular organisms (i.e., eukaryotes). Thus, considering that sexual reproduction is a nearly ubiquitous feature of eukaryotic life [19], the sexes likely represent the most consistent and widespread set of contexts over which antagonistic pleiotropy could ensue.

Although the sexes share largely the same genome, they accrue fitness in distinct ways, meaning that their fitness optima for a range of life history traits commonly differ [16–18,22]. Thus, just as Fisher’s [1] geometric model predicts the large majority of mutations to be deleterious, a rare mutation with fitness benefits in one sex will tend to pose fitness detriments in the other—the shared genome thus making SA genetic variation inevitable [20–22]. Such antagonistic mutations will then tend to reach intermediate equilibrium allele frequencies [23], while purifying selection will tend to eliminate mutations that generate SC genetic variance, leaving behind mostly SA genetic variance in fitness [24] (see Fig 1). In addition, the constraint that a shared genome poses to sex-specific adaptive evolution implies that there is only a limited extent to which SA polymorphisms can be resolved relative to the rate at which novel SA mutations occur [17–18,25–29], consistent with a growing body of empirical evidence for standing SA genetic variation (e.g., [26,30–40]). Lastly, even sex differences in the strength of selection for alternative allelic variants underlying SC forms of antagonistic pleiotropy between different components of fitness (e.g., survival, fecundity, fertility, mate competition, etc.) can render allelic trade-offs to have SA effects on overall fitness [70], suggesting that (1) the likelihood of SA genetic variation for fitness is even greater than previous theory would suggest and (2) studies that lack evidence of SA genetic variance for a given component of fitness do not speak to the presence or absence of SA genetic variation for fitness.

Though SA genetic variation for fitness may be theoretically inevitable and empirically common (see above), the extent to which it may explain fitness variance would be substantially broadened if SA polymorphisms commonly exhibited SSDR [41–42]. This would cause a net heterozygote advantage and generate stable balancing selection on those polymorphisms [41–42]. Dominance reversal between allelic variants at loci exhibiting antagonistic pleiotropy [43] was met with early skepticism (e.g., [44–46]) but is actually expected for the average SA polymorphism [47]. Further, theory predicts that SA polymorphisms favor the evolution of mechanisms that enable SSDR [48]. In support of this notion, one of the most convincing cases of a specific SA locus, albeit not for fitness per se, does indeed exhibit SSDR [39].

Our results are consistent with polygenic SSDR for the SA allelic variation underlying fitness. Using a diallel cross among isogenic strains from a well-characterized population of *C*. *maculatus* known to exhibit predominantly SA genetic variance in fitness [37–38,53,71–72], we show that the dominant–recessive relationship between alternative alleles at the loci underlying fitness was reversed in heterozygous males versus females (Fig 3). Specifically, strains whose outcrossed male fitness values tended to covary with the inbred male fitness values of the strains they were crossed with tended not to exhibit this covariance with regard to female fitness and vice versa (see Materials and methods and S6 Fig). In other words, strains whose outcrossed male fitness was determined by the strains they were crossed with (indicating those other strains’ allelic variation was dominant to their own with regard to male fitness) tended to be the determinant of female outcrossed fitness (indicating their own allelic variation was dominant to other strains’ with regard to female fitness). Thus, whether the fixed allelic variation for fitness in a given genotype tended to be dominant or recessive to that of the other genotypes (in the heterozygous progeny of crosses among them) depended upon the sex in which it was being expressed. Such SSDR will facilitate the stable maintenance of SA polymorphisms for fitness via balancing selection, enhancing their contribution to a population’s genetic variance. Indeed, this is likely a major contributor to the fact that this population exhibits predominantly SA genetic variance in fitness [37]. We repeated our test of SSDR after accounting for SC effects in the data to provide a more explicit test of SSDR for the remaining SA allelic effects, per se. The result remained highly significant but was slightly weaker (S1 Fig), which may suggest some SSDR for allelic variation with SC fitness effects (see Introduction).

The prediction that dominance deviations (the BLUPs for dominance variance) should be negatively correlated between the traits/sexes under antagonistic pleiotropy [4] was not upheld by our data (not reported). Other approaches using the dominance deviations (e.g., correlating additive breeding values and dominance deviations) were likewise ineffective at revealing the apparently strong signature of SSDR in our data (not reported). Lastly, even our extensive geometric interpretation of variance components—revealing that sex-specific dominance variance stemmed from SA dominance deviations—provided an inadequate picture of the true extent of SSDR exhibited by this population. Thus, variance partitioning and correlating dominance deviations are likely not sufficient to document dominance reversal. Future studies aiming to investigate dominance reversal via quantitative genetic methods should aim to perform full diallel crosses and assess the relative amount of dominant alleles within each strain via Hayman’s method [50,59] (see Materials and methods) so as to correlate those measures between traits, niches, and sexes that are hypothesized to be under antagonistic selection.

In addition to dominance reversal, theory has shown that epistatic interactions between SA loci can promote the maintenance of SA polymorphisms for fitness [73]. Epistasis is an expectation for polygenic traits that is derived from Fisher’s geometric model [1,73–75]. That is, because of the diminishing returns of additional beneficial alleles in increasingly beneficial backgrounds, the effect of a given allele underlying a continuous trait depends on which alleles are present at the remaining or background loci underlying that trait [73–75]. Despite it being difficult to detect empirically [74] and sometimes analyzed inappropriately [76], there is evidence of diminishing returns epistasis (e.g., [77]). However, the role of epistasis in contributing to the maintenance of SA polymorphisms for fitness has received little if any empirical attention (e.g., [78]). We found that the variance component explaining the next most phenotypic variance in fitness after dominance and SA dominance was SC epistasis. Its sex-specific counterpart was identified as describing SA epistatic effects (though its standard error overlapped zero). This represented one of the few discrepancies between our REML and Bayesian analyses, the latter identifying a sizeable contribution to fitness variance from both SC and SA epistatic effects (S3 Fig and S1 Table). However, an interpretation of the SA epistatic deviations is less straightforward than that for SA dominance deviations (see above). First, (sex-specific) epistatic deviations are a property of crosses, not strains, but the geometric interpretation of this inheritance class was based on strain means for (sex-specific) epistatic deviations (see Materials and methods). Thus, it is the crosses of some strains that tended to exhibit epistatic deviations (i.e., deviations from the expectation based on the additive contribution from each parent strain) in one sex but not the other or to different degrees in the sexes. Second, (SA) epistatic variance can be generated by a variety of interactions among loci and may not merely be due to the diminishing returns of additive loci. Thus, whether the SA epistatic effects detected are explicitly the type that could maintain genetic variance in fitness remains unclear. At most, therefore, our findings can only provide mixed evidence of a putative role for SA diminishing returns epistasis among the loci underlying fitness in this population.

As with dominance reversal, parental effects such as sex-linkage, cytoplasmic, or epigenetic effects could also partially resolve SA polymorphisms and contribute to the maintenance of SA genetic variance in fitness [25,79–83]. There is some empirical support for this (e.g., [32]), but we detected little or no variance in fitness attributable to any form of parental effects or asymmetric epistasis (i.e., parental-effects epistasis), despite the unrivaled explicit exposure of parental effects variance via the reciprocal crosses of a full diallel [50]. Although this is not to say that parental effects are nonexistent in this population, it does suggest that such effects have a relatively minor role in generating fitness variance.

Our study provides novel evidence for polygenic SSDR for the SA genetic variation underlying fitness, adding significant insights to our understanding of SA genetic variation and the maintenance of genetic variance in fitness. We hope that our findings will stimulate further efforts along these lines, which we suspect will add to the growing consensus that SA selection is a widespread phenomenon among sexually reproducing species that commonly acts to maintain genetic variance in fitness.

## Materials and methods

### Study organism

*C*. *maculatus* (Coleoptera: Bruchidae) is a pest of leguminous crops that has colonized most of the tropical and subtropical regions of the world [84]. Thus, laboratory conditions (see below) closely resemble the grain storage facilities and crop fields they have inhabited since the early Holocene. Females lay eggs on the surface of dry beans and hatched larvae bore into the beans, where they complete their life cycle, emerging from the beans as reproductively mature adults [84]. This species is facultatively aphagous (requiring neither food nor water to reproduce successfully) and exhibits a polyandrous mating system [54], X/Y sex determination [55], and pronounced sexual dimorphism [33,38] and sex-biased gene expression [56].

### Study population

The origin of our study population has been described by Berger and colleagues [37] and Grieshop and colleagues [53]. Briefly, the population was isolated from *Vigna unguiculata* seed pods collected at a small-scale agricultural field close to Lomé, Togo (06°10′N 01°13′E) during October and November 2010. Seed pods were stripped in the laboratory and beans isolated individually. Virgin males and females hatching out of these beans were paired randomly, and each pair founded an isofemale line (*n* = 41), each of which was thus derived from a single maternal and a single paternal genome. These isofemale lines were expanded and cultured at population sizes of 200–400 adults on 150 ml of *V*. *unguiculata* seeds at 29°C, 55% RH and a 12L:12D light regime for about 12 generations prior to the sex-specific fitness assays conducted by Berger and colleagues [37]. The development of isogenic (inbred) strains from these isofemale lines is described by Grieshop and colleagues [53] and in the S1 Text. The 16 inbred strains used in the present study were reasonably evenly distributed about the population’s original intersexual genetic correlation for fitness (S7 Fig) and apparently captured an unbiased representation of the standing SA genetic variation for fitness exhibited by the wild population from which it was derived (see Results and S1 Text).

### Diallel experiment

We performed a full diallel cross [50] among 16 inbred strains, generating an F_1_ generation of 240 possible outcrossed combinations and 16 parental selfs. The phenotype we measured in F_1_ individuals was sex-specific competitive lifetime reproductive success (i.e., fitness). F_1_ male (*N* = 1,731) and female (*N* = 1,547) fitness was assayed separately by placing a single focal individual in a container with approximately 25 g of *V*. *unguiculata* seeds, a sterile same-sex reference competitor (from an outbred base population established at the same time and from the same population as the isofemale lines; see above), and two opposite-sex reference beetles (a 1:1 sex ratio; see S2 Fig). Assays were placed in incubators at 29°C, 55% RH and a 12L:12D light regime until all F_2_ offspring emerged from the beans. The number of F_2_ offspring produced by an assay represents the focal (nonsterile) individual’s fitness. The sperm of sterilized males still function and fertilize eggs (but the zygotes are inviable), such that male fitness assays included pre- and postcopulatory selection (see [72] for details). Female assays also included mating competition, as well as competition for oviposition substrate (beans), and females’ ability to endure harmful repeated mating attempts by competing males in order to survive and oviposit [38,85–86]. These fitness assays not only include many aspects of the natural ecological setting for these beetles but also represent complex physical environments (i.e., the geometry of the beans), which may play an important role in enabling laboratory fitness assays to reflect complex natural environments and enable mating interactions, sexual selection, and sexual conflict to ensue more naturally [87–88].

The diallel experiment was performed twice, in two “blocks” (with cells replicated within and between blocks). In total, we performed 3,278 fitness assays (1,731 male and 1,547 female), with only moderate imbalance over the 256 families and 2 replicate blocks. Imbalance over sex, cross, and/or block categories was, however, unavoidable: different crosses (including inbred selfs) produced different numbers of F_1_ offspring in different sex ratios and had different probabilities of producing zero F_1_ offspring, providing variable opportunity to assay F_1_ fitness throughout the diallel. However, because of the large sampling effort, only 3 out of 240 outbred crosses (and 0 parental selfs) were missing from the total data set.

### REML statistical modeling

We fit a custom version of Hayman’s [59] model (modified to accommodate sex-specific data) using the FDIALLEL procedure [57] in GenStat (v.18.2.0.18409; [58]):

$$y=\mu +x+b_{1}+a+b_{2}+c+b_{3}+d+S+S\times b_{1}+S\times a+S{\times b}_{2}+S\times c+S\times b_{3}+S\times d+\varepsilon ,$$

where *μ* is the intercept for the total phenotypic variance in fitness *y*, which is partitioned into that attributable to residual error variance *ε*, the overall fixed effects of replicate block *x*, inbreeding *b*_1_, sex *S*, and sex-specific inbreeding *S*×*b*_1_, as well as the following strain- and cross-specific random effects (i.e., variance components): additive genetic variance *a*, dominance *b*_2_, parental effects *c*, symmetric epistasis *b*_3_, asymmetric epistasis *d*, and the interaction of each of those random effects with *S*. FDIALLEL forms the factors and matrices necessary to fit a diallel model using GenStat’s REML directive [57,89]—the REML directive accepting other fixed (e.g., *S*) and/or random effects (e.g., *S*×*b*_2_). The model was performed on log-transformed data, as this provided a superior model fit. This customization of Hayman’s [59] approach did not alter any of the underlying modeling of the variance components as defined in GenStat’s [58] FDIALLEL procedure [57]. We report F statistics and *P* values for the overall (fixed) effects and variances (*σ*^2^) with standard errors for the variance components (random effects). A variance component with a positive variance and standard error that excludes zero is interpreted as evidence for that mode of inheritance contributing to the observed phenotypic variance in fitness. It is possible, by this approach, to attain negative variance component estimates, which should, of course, be interpreted as not differing from zero and should not disrupt the estimation of other variance components in the model.

Replicate block (*x*) was included as an additional fixed effect because it has only two levels—i.e., too few levels to be modeled as a random effect [90]. Block had a significant effect, likely stemming from imbalance described above—the two replicate blocks differed in overall sample size and in the patterns and degree of sampling imbalance among crosses.

To aid interpretation, we also performed separate male and female models:

$$y_{M}=\mu +x+b_{1_{M}}+a_{M}+b_{2_{M}}+c_{M}+b_{3_{M}}+d_{M}+\varepsilon ,$$

and

$$y_{F}=\mu +x+b_{1_{F}}+a_{F}+b_{2_{F}}+c_{F}+b_{3_{F}}+d_{F}+\varepsilon ,$$

respectively.

### Geometric interpretation of inheritance classes

The separate male and female models enabled a geometric validation of the meaning of variance components by relating the predicted values for each strain (i.e., their BLUPs) between sexed and unsexed models as follows. The BLUPs for a given inheritance class *Q* from separate male and female models (*q*_*M*_ and *q*_*F*_) can be set as variance-standardized *y* and *x* axes, respectively (Fig 1). That coordinate system can be rotated 45° to derive BLUPs for SC (*q*_*SC*_) and SA (*q*_*SA*_) additive genetic variance (Fig 1) as done by Berger and colleagues [37] and Grieshop and colleagues [53], like so:

$${q_{SC}}_{BLUPs}={q_{F}}_{BLUPs}\sin\left(45{}^{\circ}\right)+{q_{M}}_{BLUPs}\cos\left(45{}^{\circ}\right),$$

and

$${q_{SA}}_{BLUPs}={q_{F}}_{BLUPs}\cos\left(45{}^{\circ}\right)-{q_{M}}_{BLUPs}\sin\left(45{}^{\circ}\right).$$

Note that no variance is lost during this rotation.

For many inheritance classes, the BLUPs for *q*_*SC*_ and *q*_*SA*_ (derived from separate male and female models as described above; see Fig 1) were highly correlated with the BLUPs of *q* and *S*×*q* (estimated by the full sexed model), respectively. For example, in the case of additivity, *a* was correlated to *a*_*SC*_ ($r_{a,a_{SC}}$ = 0.98, *P* < 0.0001), and *S*×*a* was correlated to *a*_*SA*_ ($r_{S\times a,a_{SA}}$ = 0.99, *P* < 0.0001). Thus, staying with the specific example of additivity,

$$a\approx a_{SC},$$

$$S\times a\approx a_{SA},$$

and

a⊥S×a,

or in words, the additive genetic variance *a* from a full sexed model represents SC additive genetic variance, the sex-specific additive genetic variance *S*×*a* represents SA additive genetic variance, and they are orthogonal to one another (see Fig 1).

Having verified that all unsexed variance components *q* from the full sexed model were modeling SC effects (see Results), a more straightforward measure of the angular relationship between two axes of variation *q* and *S*×*q* for a given inheritance class *Q* (in order to assess whether the latter represents SA variance) is their inner product *q* ∙ *S*×*q*, defined as:

$$q∙S\times q=q_{1}∙{S\times q}_{1}+q_{2}∙{S\times q}_{2}+\ldots +q_{n}∙{S\times q}_{n},$$

where q_1_…q_*n*_ and S×q_1_…S×q_*n*_ (nonitalicized) are the BLUPs of each strain for variance components *q* and *S*×*q*, respectively. Note that the inner products between sexed and unsexed variance components for symmetric and asymmetric epistasis were calculated based on strain means of BLUPs, since those variance components are based on 120 and 240 unique strain–strain combinations (in a 16 × 16 diallel), respectively.

The inner product between two axes of variation equals zero when they are orthogonal to one another (or in this case, when two variance components are describing orthogonal axes of variation). For normalized axes of variation (ours being variance-standardized prior to coordinate system rotation; see above), the inner product between *q* and *S*×*q* can be converted to the more intuitive angular displacement, *θ*, like so:

$$\theta_{q,S\times q}=\arccos(q∙S\times q).$$

We can thus calculate and verify the angular displacement of all sexed variance components from their respective unsexed/SC counterparts via their BLUPs. Note that BLUPs from variance components with negative estimates (e.g., *c* and *S*×*d*; see Results) are not valid and can therefore not be used in this geometric interpretation. The major insight gained by this exercise is that all unsexed variance components are modeling SC effects and that some sexed variance components (see Results) are modeling SA effects in this population—symmetric orthogonal deviations from the mean of their respective unsexed/SC counterparts (see Fig 1).

### Assessment of SSDR

Environmental and epistatic variance was removed from the data by taking the residuals from the following model fit, again using the FDIALLEL procedure [57] in GenStat [58] (see above):

$$y=\mu +x+b_{3}+\varepsilon ,$$

where *μ* is the intercept, *x* is the fixed effect of replicate block, *b*_3_ is a random effect modeling symmetric epistasis, and *ε* is the residual error variance. Thus, all other effects (but namely, sex-specific additive and dominance effects) remain as underlying contributions to variance in the residual data. These residuals were not variance-standardized.

Family means were tabulated from these residuals and, *σ*_*P*,*r*_, each strains’ covariance between its outcrossed family means (*r*) and the means of the respective inbred parental selfs (*P*) that correspond to each of those outcrossed families (i.e., *W*_*r*_ of Fig 1 in Hayman [59] and $\sigma_{P_{2},r}$ of Fig 20.4 in Lynch and Walsh [50]) was used as an estimate of the relative amount of recessive alleles within each strain. Each strain’s sire- and dam-specific covariances are averaged, and this was done separately for male ($\sigma_{{P_{M},r}_{M}}$) and female ($\sigma_{{P_{F},r}_{F}}$) fitness. If we denote the family mean $\bar{z}$ for a given sex of a given dam–sire combination as ${\bar{z}}_{dam,sire}$, then *σ*_*P*,*r*_ for strain 1 of that sex would be the covariance of the elements in these two vectors:

$$r_{dam}:\;({\bar{z}}_{1,2},{\bar{z}}_{1,3},{\bar{z}}_{1,4},\ldots {\bar{z}}_{1,16})$$

$$P:\;({\bar{z}}_{2,2},{\bar{z}}_{3,3},{\bar{z}}_{4,4},\ldots {\bar{z}}_{16,16}),$$

averaged with the covariance of the elements in these two vectors:

$$r_{sire}:\;({\bar{z}}_{2,1},{\bar{z}}_{3,1},{\bar{z}}_{4,1},\ldots {\bar{z}}_{16,1})$$

$$P:\;({\bar{z}}_{2,2},{\bar{z}}_{3,3},{\bar{z}}_{4,4},\ldots {\bar{z}}_{16,16}).$$

Again, this was done for each strain and for male and female fitness separately, for a total of 32 independent covariances (i.e., 16 $\sigma_{{P_{M},r}_{M}}$, and 16 $\sigma_{{P_{F},r}_{F}}$; see S6 Fig).

Strains whose outcrossed (heterozygous) means (*r*) are determined by (i.e., covary with) the inbred (homozygous) means (*P*) of the strains to which they are crossed have alleles that are apparently recessive to those of other strains, whereas strains whose heterozygous means (*r*) are independent of (i.e., do not covary with) the homozygous means (*P*) of the strains to which they are crossed have alleles that are apparently dominant to those of other strains. Thus, the correlation between $\sigma_{{P_{M},r}_{M}}$ and $\sigma_{{P_{F},r}_{F}}$ provides an indication of whether the allelic variation among strains tends to exhibit the same dominant–recessive relationship in both sexes (given by a positive correlation) or whether the dominant–recessive relationship of the allelic variation among strains is reversed between the sexes (given by a negative correlation).

Note that Hayman [59] and Lynch and Walsh [50] point out that *σ*_*P*,*r*_ and the variance among outcrossed family means *r* (i.e. *V*_*r*_ of Fig 1 in Hayman [59] and $\sigma_{r}^{2}$ of Fig 20.4 in Lynch and Walsh [50]) should scale perfectly with a regression coefficient of 1, where the intercept of that slope indicates the degree of dominance exhibited by the underlying loci (e.g., partial dominance, complete dominance, or overdominance). This is, of course, in the absence of epistatic variance, environmental variance, and substantial remaining heterozygosity in the inbred strains. We removed epistatic and environmental variance (see above), and our inbred strains appear to harbor little remaining heterozygosity (as indicated by the large inbreeding effect in our data; see Table 1 and S1 Table). However, we still found no relationship between *σ*_*P*,*r*_ and $\sigma_{r}^{2}$ (see above) with regard to male or female fitness, perhaps indicating various degrees of dominance among the underlying loci and/or unexplained environmental or epistatic variance in the residuals.

This test was repeated after removing the SC additive genetic effects (*a*) from the data by taking the residuals from the following model:

$$y=\mu +x+a+b_{3}+\varepsilon ,$$

and applying the same procedure described above, which provides a more explicit test of SSDR for the SA genetic variation, per se.

## Supporting information

S1 FigSSDR of the SA allelic variation underlying fitness.Scatterplots illustrating the observed SSDR for the SA allelic variation underlying fitness in this population (i.e., otherwise identical to Fig 3 except that SC additive genetic effects were statistically removed beforehand): (A) the relative amount of recessive allelic variation for fitness in males ($\sigma_{{P_{M},r}_{M}}\prime$) and females ($\sigma_{{P_{F},r}_{F}}\prime$) was significantly negatively correlated ($r_{\sigma_{{P_{M},r}_{M}}\prime ,\sigma_{{P_{F},r}_{F}}\prime }$ = −0.665 [95% CI −0.87 to −0.25], *P* = 0.005) across strains (*N* = 16; units reflect nonstandardized residual fitness from a model that removed environmental, epistatic, and additive genetic variance), and (B) the same relationship illustrated and analyzed as ranks (i.e., strains ranked in order of their relative dominance over one another; $r_{\sigma_{{P_{M},r}_{M}}\prime ,\sigma_{{P_{F},r}_{F}}\prime }$: −0.635, *P* = 0.0098). Strains tended to be enriched with SA allelic variation for fitness that was dominant in their heterozygous sons but recessive in their heterozygous daughters and vice versa. SA, sexually antagonistic; SC, sexually concordant; SSDR, sex-specific dominance reversal.(TIF)

S2 Fig Diagram of experimental design. Male (top right) and female (bottom right) fitness was assayed in the F_1_ individuals from all crosses (shown here as coming from an example cross between strain 2 [as dam] and strain 3 [as sire]) and was measured as the total number of F_2_ offspring emerging from these assays—i.e., the competitive lifetime reproductive success of F_1_ individuals.
(TIF)

S3 Fig Graphical representation of Bayesian variance partitioning. BayesDiallel VarPs (± 95% CIs; see S1 Text). VarP, variance projection.
(TIF)

S4 Fig Diallel plots of relative fitness. Difference between male (A) and female (B) posterior predictive means for relative fitness (i.e., fitness divided by mean outcrossed fitness per sex) in order to more easily identify patterns of inheritance among the outcrossed families (see S5 Fig for equivalent figure on the log-transformed data that corresponds to Table 1). Strains are arranged in reverse rank order of their HPD means for SA additive genetic variance (*a*_*S*_), with strain 1 being the most female beneficial/male detrimental and strain 16 being the most male beneficial/female detrimental. Pronounced SC additive effects (*a*) would be represented by strains having relatively easily identifiable vertical columns (the strain’s additive contribution as a sire) and horizontal rows (the strain’s additive contribution as a dam) with a consistent shade that does not vary (much) between males and females or with contributions from other strains (e.g., strain 9, panels A and B). Pronounced SA additive effects (*a*_*S*_) would be represented by easily identifiable patterns of *a* in one sex of a given strain with a shade toward the opposite extreme in the opposite sex of that strain (e.g., strain 1, panel A versus B). Alternatively, *a*_*S*_ can be visualized by looking at the whole population: a subtle light-to-dark and dark-to-light gradient from top left to bottom right among the outcrossed families is apparent in males (A) and females (B), respectively, since the strains are arranged in reverse rank order of their HPD means for *a*_*S*_. Disruptions to the “smoothness” of this gradient—generating a more mosaic pattern—represent the basis of variance in the different forms of dominance and epistasis: *b*, *v*, and *w*. Although parental effects (*c*) were not found to have an important contribution to fitness variance in this population, they would, in principle, appear as differences between sire- and dam-specific patterns of *a*. SA versions of any effect would, in principle, appear as its SC counterpart pattern exhibiting the opposite shade gradient between panels A and B. HPD, highest posterior density; SA, sexually antagonistic; SC, sexually concordant.
(TIF)

S5 Fig Diallel plots of log fitness. Difference between male (A) and female (B) posterior predictive means for log fitness (reflecting the analysis reported in Table 1), but otherwise identical to S4 Fig. The pronounced effects of inbreeding (*β*) render this figure mostly useful for visualizing the fixed effects of *S*, *β*, and *β*_*S*_. The similar average shade of heterozygotes between panels A and B represents no difference in mean fitness between males and females (*S*). The relative shade difference between inbred parental selfs (along the diagonal) and outbred heterozygotes represents the effect of inbreeding (*β*), and the difference in *β* between panels represents the sex-specific effect of inbreeding (*β*_*S*_), which was stronger in males. The inbreeding effects make it difficult to identify patterns of inheritance among the outcrossed families—this is more easily seen using relative fitness (see S4 Fig).
(TIF)

S6 FigDiagram explaining the array covariances used to test for SSDR.The example shown here would be for a given sex of strain 1 (whose family means are indexed as dam×sire), in which case the covariance between the elements of the two vectors (1×2, 1×3, … 1×16) and (2×2, 3×3, … 16×16)—corresponding to the family means for which the mother is from strain 1—would be averaged with the covariance between the elements of the two vectors (2×1, 3×1, … 16×1) and (2×2, 3×3, … 16×16)—corresponding to the family means for which the father is from strain 1—to give a single covariance for a given sex of strain 1. This was done for each strain, for male ($\sigma_{{P_{M},r}_{M}}$) and female ($\sigma_{{P_{F},r}_{F}}$) fitness separately after removing environmental and epistatic variance from the data (Fig 3), and then again after removing the SC additive effects as well ($\sigma_{{P_{M},r}_{M}}\prime$ and $\sigma_{{P_{F},r}_{F}}\prime$, respectively; S2 Fig). SC, sexually concordant; SSDR, sex-specific dominance reversal.(TIF)

S7 Fig Distribution of isogenic strains about the original intersexual genetic correlation for fitness. Log-transformed and variance-standardized mean male and female fitness for the isofemale lines from Berger and colleagues [37]. Filled in and circled in red are the ancestral isofemale lines from which the 16 isogenic strains of the present study were derived [53], demonstrating that the origins of the inbred strains are reasonably evenly distributed about the original intersexual genetic correlation for fitness. The data underlying this figure can be found in the Dryad digital repository, doi:10.5061/dryad.m06s2.
(TIF)

S1 Table Bayesian variance partitioning. BayesDiallel VarPs (see S1 Text) for fitness from the full sexed Bayesian model for overall (fixed) and strain- and cross-specific (random) effects, with upper and lower 95% credibility intervals, percentage of explained variance attributable to each effect, and MIPs (see S1 Text). MIP, model inclusion probability; VarP, variance projection.
(PDF)

S2 Table Separate male and female Bayesian models. BayesDiallel VarPs (see S1 Text) for separate male and female Bayesian models, for overall (fixed) and strain- and cross-specific (random) effects, with upper and lower 95% credibility intervals and percentage of explained variance attributable to each effect. VarP, variance projection.
(PDF)

S3 Table Separate male and female REML models. Results of separate male and female REML models displaying F statistics with *P* values for the overall (fixed) effects and variances (*σ*^2^) with s.e. for strain- and cross-specific (random) effects (variance components). REML, restricted maximum likelihood; s.e., standard error.
(PDF)

S1 Text Extended results and extended methods. Presentation of the results of the Bayesian analysis (and its comparison with the REML results) and further methodological information on the study population, statistical rationale, Bayesian statistical modeling, and the terminology and meaning of variance components. REML, restricted maximum likelihood.
(DOCX)

S1 Data Raw diallel data and summary data. Five sheets of data described as follows. Raw data: Full diallel data depicting the “fitness” for individuals of each “sex” (1 = female, 2 = male) in relation to the strain IDs of their fathers and mothers (“sire” and “dam,” respectively) and the replicate “block” to which each observation belongs. Fig 2: The summary data for Fig 2, depicting each variance component (“var.comp”), its “variance,” the s.e. around that variance, and the “upper” and “lower” bound of that standard error. Fig 3A and 3B: The summary data for Fig 3A and 3B, depicting “strain” IDs, male and female array covariances (“m.cov” and “f.cov,” respectively), and the rank order for those covariances (“m.cov.rank” and “f.cov.rank,” respectively). S1A and S1B Fig: The summary data for S1A and S1B Fig, depicting “strain” IDs, male and female array covariances (“m.cov” and “f.cov,” respectively), and the rank order for those covariances (“m.cov.rank” and “f.cov.rank,” respectively). S3 Fig: The summary data for S3 Fig, depicting each inheritance class (“class”), its “VarP,” and the “upper” and “lower” 95% credibility intervals. See Materials and methods. s.e., standard error.
(XLSX)

## Abbreviations

REML: restricted maximum likelihood
SA: sexually antagonistic
SC: sexually concordant
SSDR: sex-specific dominance reversal
