# Supplementary figures and images for: Sex-specific dominance reversal of genetic variation for fitness
Source: PLoS Biol. 2018 Dec 11;16(12):e2006810. doi: 10.1371/journal.pbio.2006810 (PMC6303075; doi:10.1371/journal.pbio.2006810)

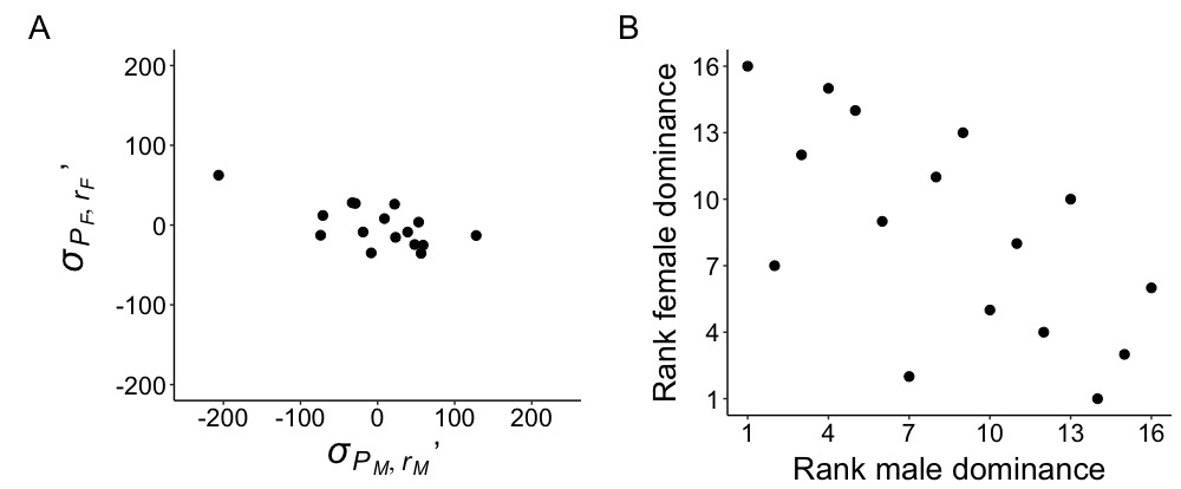

Supplement: S1 Fig — Scatterplots illustrating the observed SSDR for the SA allelic variation underlying fitness in this population (i.e., otherwise identical to Fig 3 except that SC additive genetic effects were statistically removed beforehand): (A) the relative amount of recessive allelic variation for fitness in males (σPM,rM′) and females (σPF,rF′) was significantly negatively correlated (rσPM,rM′,σPF,rF′ = −0.665 [95% CI −0.87 to −0.25], P = 0.005) across strains (N = 16; units reflect nonstandardized residual fitness from a model that removed environmental, epistatic, and additive genetic variance), and (B) the same relationship illustrated and analyzed as ranks (i.e., strains ranked in order of their relative dominance over one another; rσPM,rM′,σPF,rF′: −0.635, P = 0.0098). Strains tended to be enriched with SA allelic variation for fitness that was dominant in their heterozygous sons but recessive in their heterozygous daughters and vice versa. SA, sexually antagonistic; SC, sexually concordant; SSDR, sex-specific dominance reversal. (TIF) [file pbio.2006810.s001.tif]

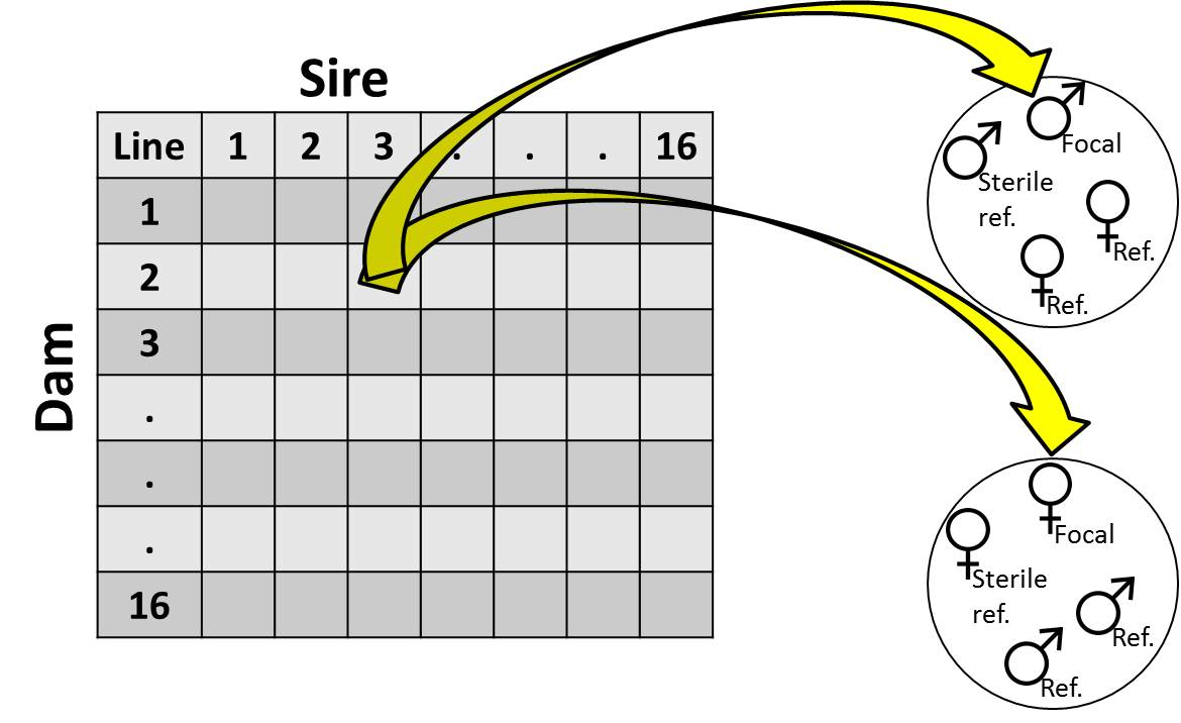

Supplement: S2 Fig — Male (top right) and female (bottom right) fitness was assayed in the F1 individuals from all crosses (shown here as coming from an example cross between strain 2 [as dam] and strain 3 [as sire]) and was measured as the total number of F2 offspring emerging from these assays—i.e., the competitive lifetime reproductive success of F1 individuals. (TIF) [file pbio.2006810.s002.tif]

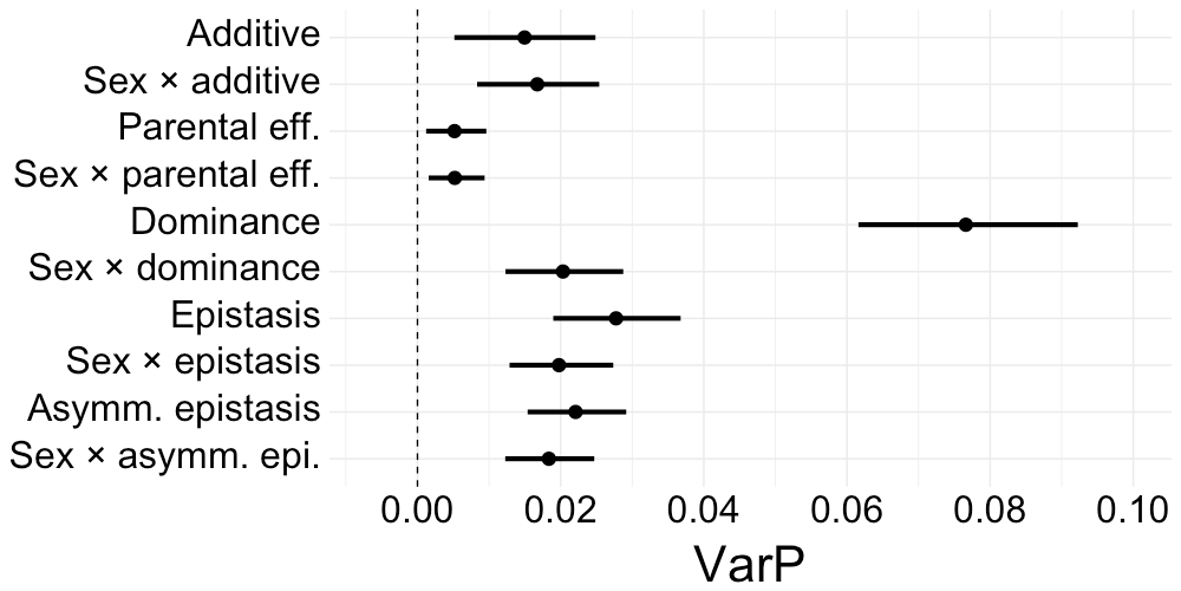

Supplement: S3 Fig — BayesDiallel VarPs (± 95% CIs; see S1 Text). VarP, variance projection. (TIF) [file pbio.2006810.s003.tif]

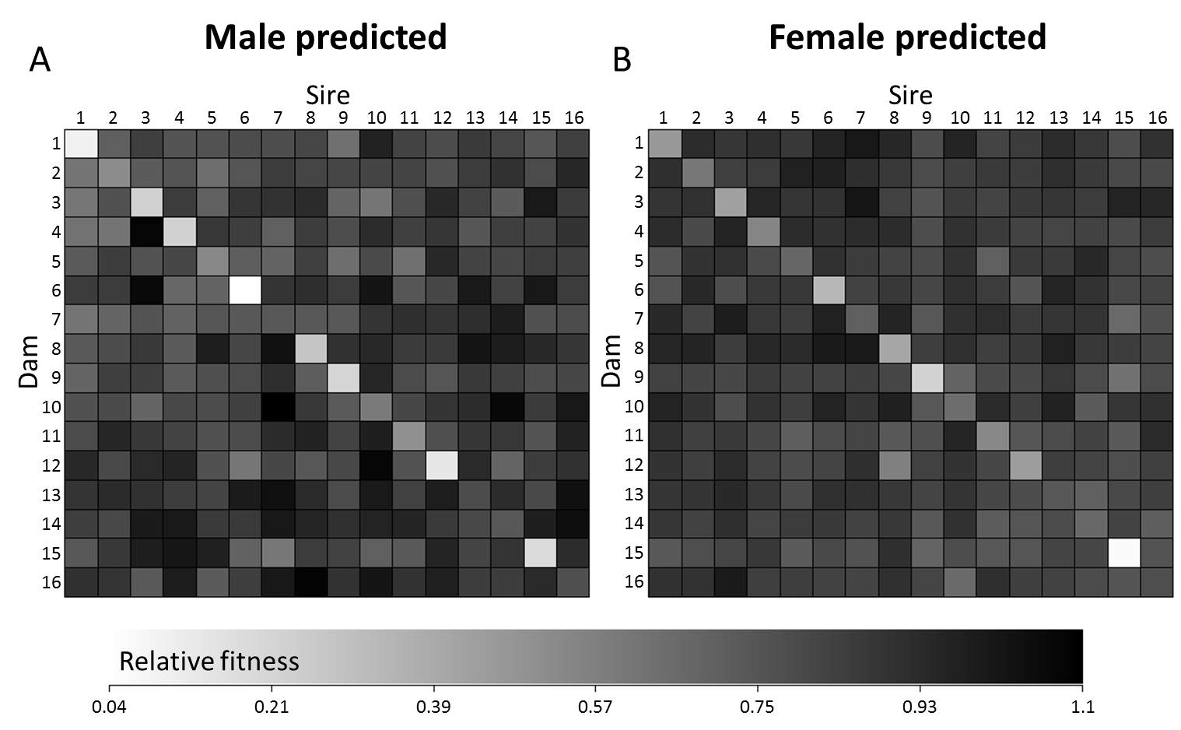

Supplement: S4 Fig — Difference between male (A) and female (B) posterior predictive means for relative fitness (i.e., fitness divided by mean outcrossed fitness per sex) in order to more easily identify patterns of inheritance among the outcrossed families (see S5 Fig for equivalent figure on the log-transformed data that corresponds to Table 1). Strains are arranged in reverse rank order of their HPD means for SA additive genetic variance (aS), with strain 1 being the most female beneficial/male detrimental and strain 16 being the most male beneficial/female detrimental. Pronounced SC additive effects (a) would be represented by strains having relatively easily identifiable vertical columns (the strain’s additive contribution as a sire) and horizontal rows (the strain’s additive contribution as a dam) with a consistent shade that does not vary (much) between males and females or with contributions from other strains (e.g., strain 9, panels A and B). Pronounced SA additive effects (aS) would be represented by easily identifiable patterns of a in one sex of a given strain with a shade toward the opposite extreme in the opposite sex of that strain (e.g., strain 1, panel A versus B). Alternatively, aS can be visualized by looking at the whole population: a subtle light-to-dark and dark-to-light gradient from top left to bottom right among the outcrossed families is apparent in males (A) and females (B), respectively, since the strains are arranged in reverse rank order of their HPD means for aS. Disruptions to the “smoothness” of this gradient—generating a more mosaic pattern—represent the basis of variance in the different forms of dominance and epistasis: b, v, and w. Although parental effects (c) were not found to have an important contribution to fitness variance in this population, they would, in principle, appear as differences between sire- and dam-specific patterns of a. SA versions of any effect would, in principle, appear as its SC counterpart pattern exhibiting the opposite sha [file pbio.2006810.s004.tif]

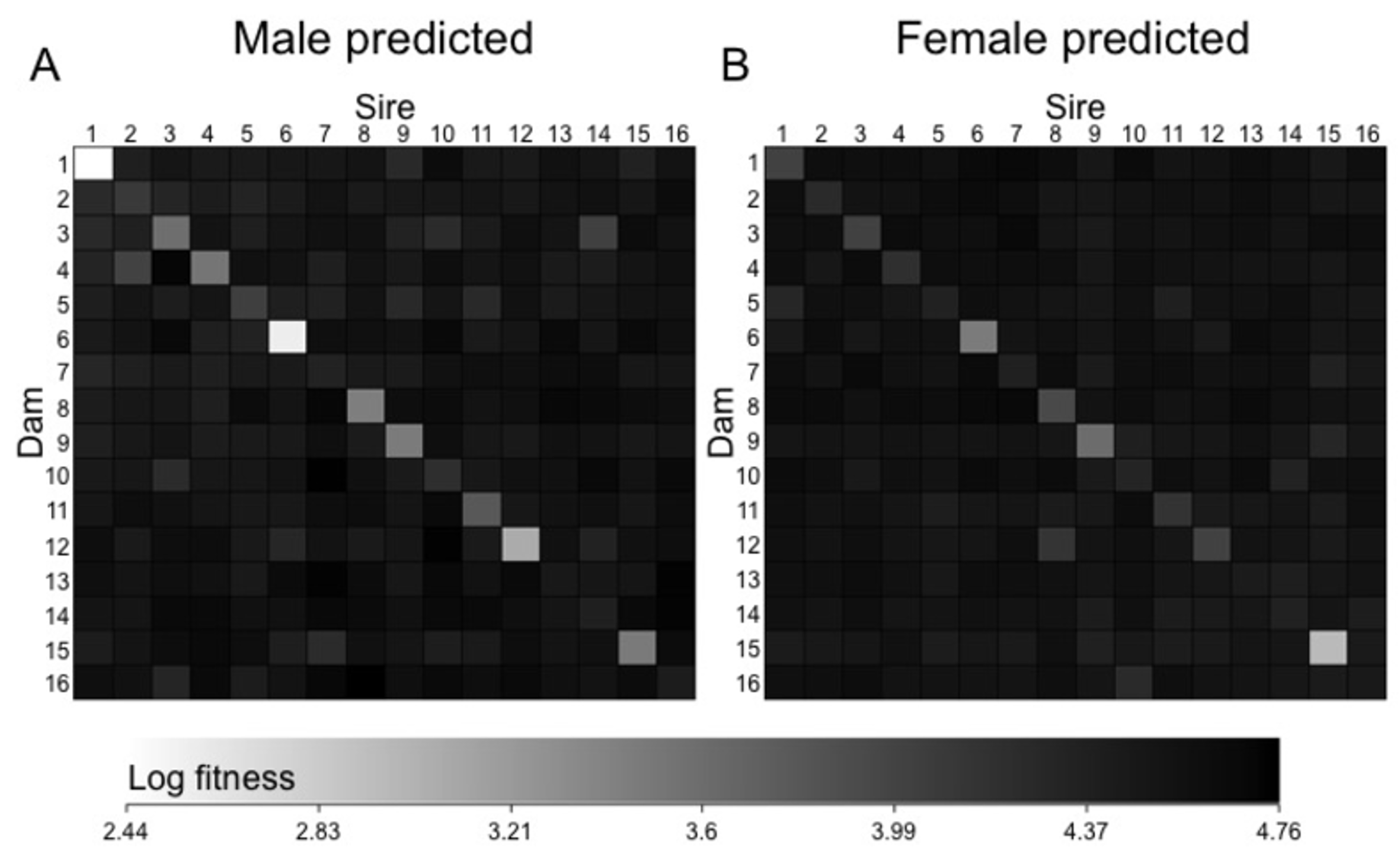

Supplement: S5 Fig — Difference between male (A) and female (B) posterior predictive means for log fitness (reflecting the analysis reported in Table 1), but otherwise identical to S4 Fig. The pronounced effects of inbreeding (β) render this figure mostly useful for visualizing the fixed effects of S, β, and βS. The similar average shade of heterozygotes between panels A and B represents no difference in mean fitness between males and females (S). The relative shade difference between inbred parental selfs (along the diagonal) and outbred heterozygotes represents the effect of inbreeding (β), and the difference in β between panels represents the sex-specific effect of inbreeding (βS), which was stronger in males. The inbreeding effects make it difficult to identify patterns of inheritance among the outcrossed families—this is more easily seen using relative fitness (see S4 Fig). (TIF) [file pbio.2006810.s005.tif]

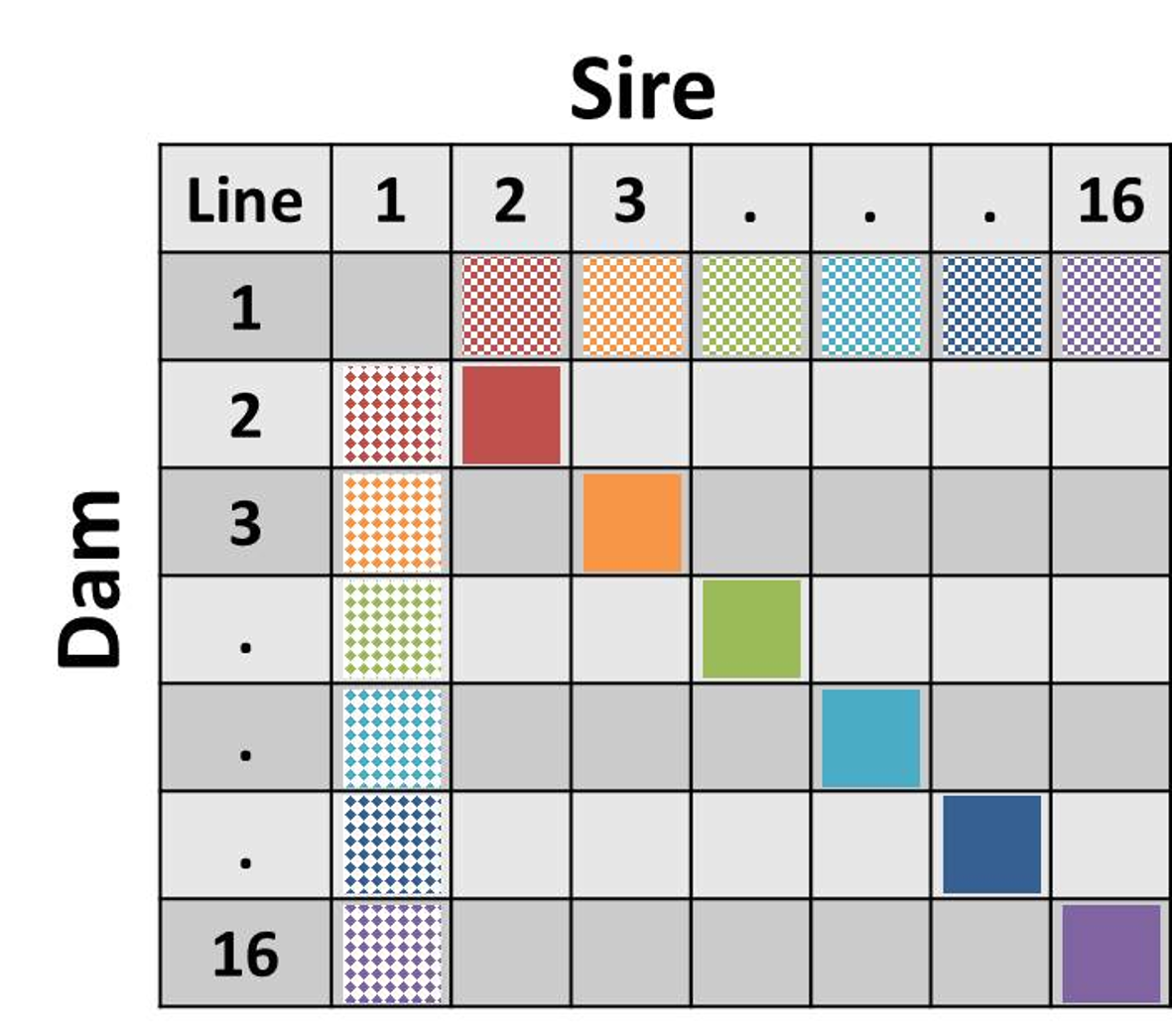

Supplement: S6 Fig — The example shown here would be for a given sex of strain 1 (whose family means are indexed as dam×sire), in which case the covariance between the elements of the two vectors (1×2, 1×3, … 1×16) and (2×2, 3×3, … 16×16)—corresponding to the family means for which the mother is from strain 1—would be averaged with the covariance between the elements of the two vectors (2×1, 3×1, … 16×1) and (2×2, 3×3, … 16×16)—corresponding to the family means for which the father is from strain 1—to give a single covariance for a given sex of strain 1. This was done for each strain, for male (σPM,rM) and female (σPF,rF) fitness separately after removing environmental and epistatic variance from the data (Fig 3), and then again after removing the SC additive effects as well (σPM,rM′ and σPF,rF′, respectively; S2 Fig). SC, sexually concordant; SSDR, sex-specific dominance reversal. (TIF) [file pbio.2006810.s006.tif]

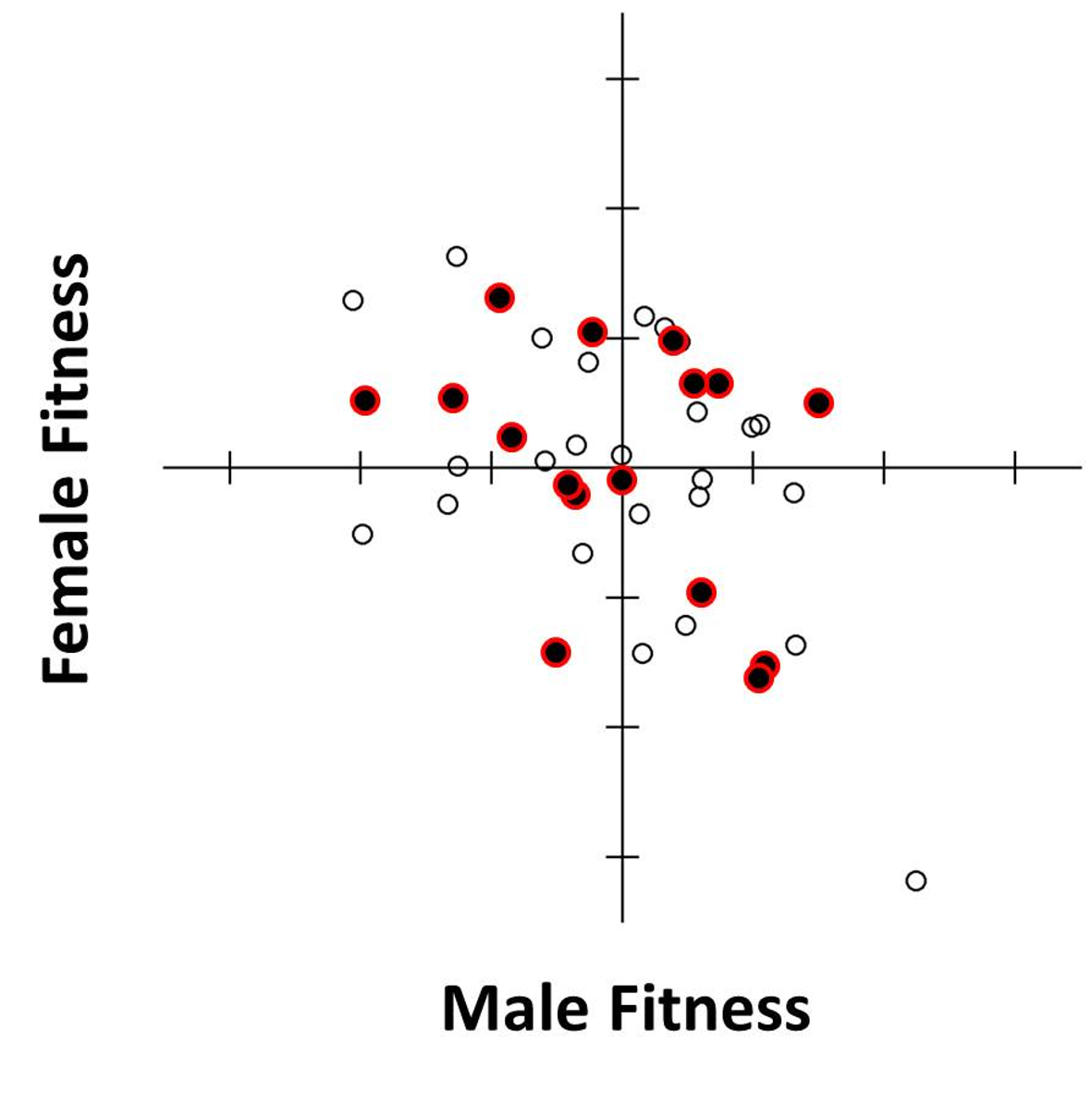

Supplement: S7 Fig — Log-transformed and variance-standardized mean male and female fitness for the isofemale lines from Berger and colleagues [37]. Filled in and circled in red are the ancestral isofemale lines from which the 16 isogenic strains of the present study were derived [53], demonstrating that the origins of the inbred strains are reasonably evenly distributed about the original intersexual genetic correlation for fitness. The data underlying this figure can be found in the Dryad digital repository, doi:10.5061/dryad.m06s2. (TIF) [file pbio.2006810.s007.tif]
