# Supplementary material for: Sex-specific dominance reversal of genetic variation for fitness
Source: PLoS Biol. 2018 Dec 11;16(12):e2006810. doi: 10.1371/journal.pbio.2006810 (PMC6303075; doi:10.1371/journal.pbio.2006810)
Supplement: S1 Table — BayesDiallel VarPs (see S1 Text) for fitness from the full sexed Bayesian model for overall (fixed) and strain- and cross-specific (random) effects, with upper and lower 95% credibility intervals, percentage of explained variance attributable to each effect, and MIPs (see S1 Text). MIP, model inclusion probability; VarP, variance projection. (PDF) [file pbio.2006810.s008.pdf]

S1 Table.

	Effect	Symbol	VarP	L. 95% C.I.	U. 95% C.I.	% of Total explained	MIP
**Overall**	Sex	S	0.001	-0.0002	0.0032	0.31	0.70
	Inbreeding	β	0.147	0.1282	0.1652	37.99	0.72
	Sex × inbreeding	β_S	0.011	0.0053	0.0167	2.86	0.71
	Block	x	0.000	0.0000	0.0000	0.00	1.00
**Strain-/cross-specific**	Additive	a	0.015	0.0052	0.0249	3.88	0.70
	Sex × additive	a_S	0.017	0.0083	0.0254	4.34	0.67
	Parental eff.	m	0.005	0.0012	0.0096	1.34	0.65
	Sex × parental eff.	m_S	0.005	0.0016	0.0094	1.35	0.09
	Dominance	b	0.077	0.0616	0.0923	19.86	0.84
	Sex × dominance	b_S	0.020	0.0123	0.0288	5.27	0.88
	Epistasis	v	0.028	0.0190	0.0368	7.19	0.88
	Sex × epistasis	v_S	0.020	0.0129	0.0274	5.12	0.79
	Asymm. epistasis	w	0.022	0.0154	0.0292	5.73	0.87
	Sex × asymm. epi.	w_S	0.018	0.0123	0.0247	4.76	0.65
	Total explained		0.386	0.3623	0.4090		
	Total unexplained		0.614	0.5910	0.6377		
