# Supplementary material for: Sex-specific dominance reversal of genetic variation for fitness
Source: PLoS Biol. 2018 Dec 11;16(12):e2006810. doi: 10.1371/journal.pbio.2006810 (PMC6303075; doi:10.1371/journal.pbio.2006810)
Supplement: S2 Table — BayesDiallel VarPs (see S1 Text) for separate male and female Bayesian models, for overall (fixed) and strain- and cross-specific (random) effects, with upper and lower 95% credibility intervals and percentage of explained variance attributable to each effect. VarP, variance projection. (PDF) [file pbio.2006810.s009.pdf]

S2 Table.

	Effect	Symbol	Female VarP	Female L. 95% C.I.	Female U. 95% C.I.	Female % of Total explained	Male VarP	Male L. 95% C.I.	Male U. 95% C.I.	Male % of Total explained
Over-all	Inbreeding	β	0.201	0.1735	0.2281	37.83	0.154	0.1279	0.1799	44.61
	Block	x	0.000	0.0000	0.0000	0.00	0.000	0.0000	0.0000	0.00
Strain-/cross-specific	Additive	a	0.049	0.0252	0.0744	9.24	0.028	0.0132	0.0435	8.14
	Parental eff.	m	0.011	0.0021	0.0197	1.99	0.010	0.0036	0.0180	2.91
	Dominance	b	0.142	0.1154	0.1682	26.66	0.090	0.0681	0.1129	26.09
	Epistasis	v	0.071	0.0499	0.0913	13.29	0.034	0.0215	0.0480	9.91
	Asymm. epistasis	w	0.058	0.0430	0.0751	10.99	0.029	0.0184	0.0397	8.34
	Total explained		0.531	0.5013	0.5623		0.345	0.3125	0.3794	
	Total unexplained		0.469	0.4377	0.4987		0.655	0.6206	0.6875	
