# Supplementary material for: Sex-specific dominance reversal of genetic variation for fitness
Source: PLoS Biol. 2018 Dec 11;16(12):e2006810. doi: 10.1371/journal.pbio.2006810 (PMC6303075; doi:10.1371/journal.pbio.2006810)
Supplement: S3 Table — Results of separate male and female REML models displaying F statistics with P values for the overall (fixed) effects and variances (σ2) with s.e. for strain- and cross-specific (random) effects (variance components). REML, restricted maximum likelihood; s.e., standard error. (PDF) [file pbio.2006810.s010.pdf]

S3 Table.

	Effect	Symbol	Female		Male	
			**F**	***P***	**F**	***P***
**Over-all**	Inbreeding	b_1	22.02	<0.001	26.00	<0.001
	Block	x	72.17	<0.001	43.79	<0.001
			$\boldsymbol{\sigma^2}$	**s.e.**	$\boldsymbol{\sigma^2}$	**s.e.**
**Strain-/cross-specific**	Additive	a	0.0026	0.0051	0.0058	0.0119
	Parental eff.	c	-0.0003	0.0003	0.0003	0.0009
	Dominance	b_2	0.0165	0.0064	0.0384	0.0149
	Epistasis	b_3	0.0012	0.0008	0.0029	0.0019
	Asymm. epistasis	d	0.0024	0.0011	-0.0060	0.0024
	Error	ε	0.0300	0.0012	0.1540	0.0057
