# Supplementary material for: Sex-specific dominance reversal of genetic variation for fitness
Source: PLoS Biol. 2018 Dec 11;16(12):e2006810. doi: 10.1371/journal.pbio.2006810 (PMC6303075; doi:10.1371/journal.pbio.2006810)
Supplement: S1 Text — Presentation of the results of the Bayesian analysis (and its comparison with the REML results) and further methodological information on the study population, statistical rationale, Bayesian statistical modeling, and the terminology and meaning of variance components. REML, restricted maximum likelihood. (DOCX) [file pbio.2006810.s011.docx]

**Extended results**

All *unsexed* terms from the full sexed Bayesian model were identified as likewise modeling SC effects (see Materials and Methods). Almost all angular displacements ($\theta$) between those SC terms and their respective *sexed* counterparts were ≈90° (see Materials and Methods), except for that between parental effects and sex-specific parental effects ($\theta_{a,a_{S}}$ = 90.50°, $\theta_{m,m_{S}}$ = 127.04°, $\theta_{b,b_{S}}$ = 88.82°, $\theta_{v,v_{S}}$ = 90.07°, and $\theta_{w,w_{S}}$ = 90.05°).

*Contrasting Bayesian and REML variance partitioning*

Both Bayesian and REML analyses revealed no overall difference between the sexes ($S$) in mean fitness (Table 1, 2). The overall effect of inbreeding ($\beta$ and $b_{1}$) was large, accounting for nearly 40% of the explained variation in the Bayesian analysis (S1 Table) – a statistically significant effect in the REML analysis (Table 1). The sexes differed in this regard; sex-specific inbreeding accounted for almost 3% of the explained phenotypic variance in the Bayesian analysis ($\beta_{S}$; S1 Table) – a significant effect in the REML analysis ($S\times b_{1}$; Table 1). Separate male and female Bayesian and REML models agreed that the effect of inbreeding was stronger in males than females (S1 Table and Table 1, respectively). Again, note that these sizeable inbreeding and sex-specific inbreeding effects have been accounted for as overall (fixed) effects prior to/alongside estimation of random effects, and therefore do not inflate or disrupt estimates of SC dominance ($b$ and $b_{2}$), SA dominance ($b_{S}$ and $S\times b_{2}$) or any other inheritance class.

In general, both Bayesian (S3 Fig) and REML (Fig 1) analyses revealed phenotypic variance in fitness to be characterized by pronounced SC dominance ($b$ and $b_{2}$), SA dominance ($b_{S}$ and $S\times b_{2}$) and epistasis ($v$ and $b_{3}$), whereas parental effects ($m$ and $c$), sex-specific parental effects ($m_{S}$ and $S\times c$) and sex-specific asymmetric epistasis ($w_{S}$ and $S\times d$) contributed relatively little or nothing (taking into account the MIPs for $w_{S}$, see below) to phenotypic variance in fitness (S1 Table and Table 1, respectively). The approaches differed slightly in the extent to which they attributed phenotypic variance to SC additive effects ($a$), SA additive effects ($a_{S}$ versus $S\times a$), SA epistasis ($v_{S}$ versus ${S\times b}_{3}$), and SC asymmetric epistasis ($w$ versus $d$; S1 Table/S3 Fig and Table 1/Fig 2, respectively) – details below.

*Bayesian-specific variance partitioning*

The Bayesian approach identified evidence of a contribution to variance in fitness from all inheritance classes, but their relative contributions varied drastically, and the model inclusion probabilities (MIPs; see below) indicate that only $b$, $b_{S}$, $v$, $v_{S}$, and $w$ exhibit positive evidence (i.e. an MIP >0.75, see below) of their importance to the model fit (S1 Table, S3 Fig).

SC dominance variance ($b$) accounted for nearly 20% of the explained phenotypic variance in fitness, with SA dominance variance ($b_{S}$) accounting for >5% (S1 Table). SC epistatic variance ($v$) and SA epistatic variance ($v_{S}$) together accounted for >12% of the explained phenotypic variance in fitness (S1 Table). SC ($w$) and SA ($w_{S}$) asymmetric epistatic variance together accounted for >10% of the explained phenotypic variance (but the later had a MIP of <0.75; S1 Table).

SC and SA additive genetic variance ($a$ and $a_{S}$, respectively) each accounted for ~5% of the explained phenotypic variance for fitness, with the VarP equivalent of SC and SA narrow sense heritability (see S1 Text) being 0.015 and 0.017, respectively (S1 Table). However, both had MIP of <0.75 (S1 Table; see S1 Text). SC and sex-specific parental effects variance ($m$ and $m_{S}$) together accounted for <3% of the explained phenotypic variance for fitness, with the former having an MIP <0.75 and the latter having a MIP of <0.1 (i.e. positive evidence of its *un*importance to model fit; S1 Table).

Consistent with the REML analysis (see Results), separate male and female unsexed models reveal, firstly, that much more of the phenotypic variance in fitness can be explained in females relative to males (with $a_{F}$ nearly 2x $a_{M}$), and secondly, that the sexes exhibit similar proportional variance assigned to each of the inheritance classes, with minor exceptions (S2 Table).

S4 and S5 Figs are diallel plots displaying the difference between male and female posterior predictive family means for relative fitness (i.e. fitness divided by mean outbred fitness per sex) and log-transformed fitness (i.e. the actual analysis reported in S1 Table), respectively. They are useful for visualizing patterns of inheritance (S4 Fig), and the effects of sex ($S$), inbreeding ($\beta$) and sex-specific inbreeding ($\beta_{S}$; S5 Fig).

**Extended methods**

*Study population*

The fitness assays performed by Berger *et al*. [1] revealed that this wild-caught population was dominated by SA additive genetic variance in fitness (r_MF_ = -0.51; [1]). Despite significant additive genetic variance for female fitness, this was not significant for *male* fitness due to environmental variance in the assays of Berger *et al.* [1]. However, male fitness was assayed again 10 generations later and shown to be highly repeatable (r = 0.80, n = 10, P <0.001; [2]), demonstrating significant genetic variance also for male fitness and confirming this population’s significant SA genetic variance in fitness. These isofemale lines were cultured for ~24 generations after their establishment prior to further inbreeding.

The development of isogenic/inbred strains from these isofemale lines was described in Grieshop *et al.* [2]. Briefly, 20 replicate lineages of each of the 41 isofemale lines (n≈800) were subject to single-pair full-sib inbreeding for 10 consecutive generations or until going extinct [2]. This protocol was intended to prevent (or at least minimize) any non-random extinction/loss of genetic variation exhibited by the wild population (i.e. captured by the 41 isofemale lines). Grieshop *et al.* [2] showed this effort was necessary, since lineages derived from male-benefit/female-detriment isofemale lines, and to a lesser extent generally low-fitness (i.e. low-male/low-female) isofemale lines, were significantly more likely to go extinct prior to completing 10 consecutive generations of inbreeding. In extreme cases, all 20 replicate lineages of some of the most male-benefit and low-fitness isofemale lines went extinct, leaving no remaining isogenic strains to represent those isofemale lines [2]. The implications of these results were discussed in depth by Grieshop *et al.* [2]. Of present concern, the effort to avoid a biased genetic architecture for fitness in this panel of inbred strains was successful in that the 16 inbred strains randomly chosen from the (n=182) remaining/extant lineages (such that no two isogenic strains came from the same isofemale line) were reasonably evenly distributed about the population’s original intersexual genetic correlation for (raw log-transformed) fitness (S7 Fig).

In terms of interpreting the precise magnitude of the different sources of variance in fitness, there may still be the potential caveat that lineages from male-benefit/female-detriment isofemale lines exhibited greater levels of extinction during the inbreeding process that was used to create the isogenic strains of the present study ([2] see above). For example, it is not surprising that there were rather similar amounts of SC and SA additive genetic variance in the synthetic diallel version of this population compared to the predominantly SA additive genetic variance in fitness observed by Berger *et al.* [1] among the isofemale lines from which it originates (see above). Importantly, however, males and females of the present study did not differ in their fitness relative to reference competitors – i.e. the fixed effect of ‘sex’ was non-significant – suggesting that while our panel of 16 isogenic strains may have exhibited proportionally *less* SA genetic variance in fitness than its ancestral population, it nevertheless captured an unbiased/non-skewed representation of that original standing SA genetic variance. This notion is also supported, less directly, by the relatively even distribution of the isogenic line origins throughout this population’s original intersexual genetic correlation for fitness (S7 Fig).

*Statistical rationale*

There are several ways to analyze diallel data [e.g. 3-7]. Most of these approaches are less than ideal or inappropriate for the present data due to one or more of the following reasons: 1) treating parental strains as fixed effects – as the aim of the present study was to characterize the genetic architecture of a population as represented by a panel of randomly chosen inbred parental strains, strains and inheritance classes should be modeled as random effects [7]; 2) the inability to estimate dominance variance [7]; 3) the inability to estimate sex-specific variance components – modeling sex-specific variance components not only doubles the number of parameters in the model but can also change the meaning of other parameters depending on the method [6]; and/or 4) the inability to handle imbalanced data and/or empty cells – due to the statistical complexity of diallel analyses, the results of conventional ANOVA performed on imbalanced data can be biased and/or unreliable [7(pp. 627),6].

Two general approaches are used to address the fourth issue: restricted maximum likelihood (REML) estimation and Bayesian simulation [7,6]. Lenarcic *et al*.’s [6] hierarchical Bayesian approach is, however, the only method that explicitly addresses all four of the above issues. It was developed with the intention of modeling sex-specific parameters, and automatically incorporates uncertainty due to imbalance, empty cells, and small sample size into its estimates [6]. We used Lenarcic *et al*.’s [6] model as a template upon which to base our custom REML parameterization (see Materials and Methods) with the help of GenStat’s [8] support team (see Acknowledgements), which was a version Hayman’s [3] approach that we modified to accommodate sex-specific data. The Bayesian analysis largely validated our custom REML fit (see above). It was desirable in our case to have the REML model be the focus of our analysis, since a sub-set of that REML model served as the basis for our assessment of sex-specific dominance reversal (see Materials and Methods), which was the focus of our study. For clarity, we have used uniform terminology across the two approaches. For further details regarding similarities and differences in the modeling and/or terminology between approaches, see Hayman [3], Lenarcic *et al*. [6], Crowley *et al*. [9], and Payne [10].

*Bayesian Statistical modeling*

For the Bayesian approach we fit Lenarcic *et al.*’s [6] full sexed (‘fulls’) model using the ‘BayesDiallel’ MCMC Gibbs sampler [11] for R (v.3.2.1; [12]):

$$y=\mu+x+\beta+a+b+m+v+w+S+\beta_{S}+a_{S}+b_{S}+m_{S}+v_{S}+w_{S}+\varepsilon,$$

where $\mu$ is the intercept for the total phenotypic variance in fitness $y$, which is partitioned into that attributable to residual error variance $\varepsilon$, the overall fixed effects of replicate block $x$, inbreeding $\beta$, sex $S$, and sex-specific inbreeding $\beta_{S}$, and the strain-/cross-specific random effects (i.e. inheritance classes): additive genetic variance $a$, dominance $b$, parental effects $m$, symmetric epistasis $v$, asymmetric epistasis $w$, and sex-specific random effects (subscripted with ‘S’). We ran 5 chains for 11000 iterations, with the first 1000 discarded as a burn-in – more than enough to achieve unimodal, symmetric posteriors. We achieved the greatest proportion of variance explained when setting the ‘LogTransform’ and ‘DoFirstCenter’ arguments to ‘TRUE’, which log-transforms the response variable and mean-centers the chains, respectively.

The Bayesian parameter estimates reported for each inheritance class are diallel variance projections (VarP), following Crowley *et al.* [9]. This approach uses the posterior predictive distribution of the effects parameters of each strain to simulate perfectly balanced, complete, future diallels of the same parental strains. Then, for each of those simulated data sets, it divides each inheritance class’ sum of squares by the overall phenotypic sum of squares, which provides a posterior distribution of proportions for each inheritance class, with a highest posterior density mean (i.e. VarP) and upper/lower 95% credibility intervals [9]. In the case of the additive inheritance class, this proportion is similar to narrow-sense heritability [7]; however, its estimation is said to be more precise because it is based on the effects parameters of each strain rather than the variance components, and is therefore more directly informed by the data [9]. An inheritance class with 95% credibility intervals excluding zero is interpreted as strong evidence for a given inheritance class’ contribution to the observed phenotypic variance. No VarP estimate had credibility intervals below zero, raising the suspicion that these estimates may have been biased upward, or constrained to be positive. However, as mentioned above, the posterior distributions for all inheritance classes were symmetrical, indicating they were not constrained to be positive.

In addition, we report the model inclusion probabilities (MIPs) for each inheritance class from the accompanying ‘BayesSpike’ model selection procedure of the BayesDiallel package [9,11]. MIPs near 1 indicate a term being essential to the model fit, near 0 indicate a term having a negligible effect on the model fit, and near 0.5 indicate a term’s importance to the model fit is uncertain given the data collected so far [6,9]. The interpretation of BayesSpike MIPs approximately follows that corresponding to Kass and Raftery’s [13] interpretation of Bayes factors: those in the range of (0.75, 0.95), (0.95, 0.99), and (0.99, 1) respectively representing positive, strong and very strong evidence for a term’s importance to the model fit, and those in the range of (0.05, 0.25), (0.01, 0.05), and (0, 0.01) respectively representing positive, strong and very strong evidence for a term’s *un*importance to the model fit [6,9].

As in the REML analysis, separate male and female Bayesian models were performed to aid interpretation:

$$y_{M}=\mu+x+\beta_{M}+a_{M}+b_{M}+m_{M}+v_{M}+w_{M}+\varepsilon,$$

and

$$y_{F}=\mu+x+\beta_{F}+a_{F}+b_{F}+m_{F}+v_{F}+w_{F}+\varepsilon,$$

respectively. This also provided the means by which to perform the geometric interpretation of *sexed* and *unsexed* terms from the full sexed model, which was based on highest posterior density (HPD) means in the case of the Bayesian analysis (the Bayesian equivalent of BLUPs).

*Terminology and meaning of variance components*

Here, we explain the most likely causal phenomena underlying each variance component in familiar terms, with the aim to aid the interpretation of our results. We may use SC or SA as defined in Fig 1, where appropriate, according to our geometric interpretation of inheritance classes (see Results). As a reminder, we are using uniform terminology across the two approaches, for clarity. For details regarding modeling and/or terminology, see Hayman [3], Lenarcic *et al*. [6], Crowley *et al*. [8], and Payne [9].

Additive genetic variance ($a$) is based on the effect that strains have on their offspring regardless of whether they serve as the mother or the father, and regardless of which other strains they are crossed with, revealing the extent to which variation in fitness is attributable to the SC additive effects of autosomal alleles. Sex-specific additive genetic variance ($a_{S}$ and $S\times a$) reveals the extent to which variation in fitness is attributable to SA additive effects of autosomal alleles.

Parental effects variance ($m$ and $c$) is based on sire-dam asymmetry in the additive effects of reciprocal crosses. Assuming the parental strains are completely homozygous such that reciprocal full sibs effectively have the same parents (autosomally) but the sex of those parents is reversed so that the father of one sibling is the mother of the other, and vice versa, and such that reciprocal half sibs effectively have one parent in common but that parent is the father of one sibling and the mother of the other [7], then sire-dam asymmetry in the SC additive effects reveals the extent to which variation in fitness is attributable to the SC effects of parental contributions to zygotes that differ between mothers and fathers (e.g. sex-linked variation, mitochondrial variation, or variation in epigenetic imprinting). Sex-specific parental effects variance ($m_{S}$ and $S\times c$) reveals the extent to which variation in fitness is attributable to parental contributions to zygotes that affect males and females differently, but are not necessarily SA effects according to the findings in our data (see Results and Discussion; e.g. variation in sex-biased gene expression due to sex-chromosome variation, sex-specific effects of mitochondrial variation, or sex-specific epigenetic imprinting).

For dominance variance, $b$ and $b_{2}$ are based on different predictive baselines ($\beta$ and $b_{1}$: the “inbreeding penalty” and “mean dominance deviation” of Lenarcic *et al.* [6] and Hayman [3], respectively). Both $b$ and $b_{2}$ reveal the extent to which variation in fitness is attributable to the way in which strains’ ‘inbreeding’ effects ($\beta$ and $b_{1}$) – i.e. homozygous versus heterozygous values – vary over the different strains to which they are crossed. Assuming the parental strains are completely homozygous such that all loci in the F_1_ *population* are heterozygous with allele frequencies = 0.5 [7], variance in $b$ and $b_{2}$ stem from SC deviations from the SC additive expectation that ultimately due to varying dominant/recessive relationships among strains’ SC additive alleles (i.e. from the older literature, the so-called ‘within-locus’ epistasis). This therefore reveals the extent to which variation in fitness is attributable to SC dominance relationships between autosomal allelic variants. Sex-specific dominance variance ($b_{S}$ and $S\times b_{2}$) reveals variation in fitness attributable SA dominance deviations between autosomal allelic variants.

Symmetric epistatic variance ($v$ and $b_{3}$) is based on the statistical interaction between strains – i.e. how a strains’ effects on their offspring depend the strain to which they are crossed (it can be thought of as a factor with 120 levels in a 16 × 16 diallel, because sire-dam differences are not considered). This reveals the extent to which variation in fitness is attributable to interactions among autosomal allelic variants, which produce SC effects. Sex-specific epistatic variance ($v_{S}$ and ${S\times b}_{3}$) reveals the extent to which variation in fitness is attributable to interactions among autosomal allelic variants, which produce SA effects.

Asymmetric epistatic variance ($w$ and $d$) is based on sire-dam asymmetry in the epistatic effects (and could be thought of, alternatively, as *parental-effects epistasis*; or a factor with 240 levels in a 16 × 16 diallel). This reveals the extent to which variation in fitness is attributable to interactions among parental contributions (e.g. variation in the effects of certain mitochondrial/sex-chromosome combinations), as well as interactions between parental contributions and autosomal variation (e.g. variation in the effects of certain sex-chromosome/autosome combinations, or certain mitochondrial/autosome combinations), which produce SC effects. Sex-specific asymmetric epistatic variance ($w_{S}$ and $S\times d$) reveals the extent to which variation in fitness is attributable to interactions among parental contributions (e.g. variation due to SA effects of certain mitochondrial/sex-chromosome combinations), as well as interactions between parental contributions and autosomal variation (e.g. variation in sex-biased gene expression attributable to certain sex-chromosomes/autosome combinations, or variation due to SA effects of certain mitochondrial/autosome combinations), which produce SA effects. **References for S1 Text**

1. Berger D, Grieshop K, Lind MI, Goenaga J, Maklakov AA, Arnqvist G. Intralocus sexual conflict and environmental stress. Evolution. 2014 Aug 1;68(8):2184-96.
2. Grieshop K, Berger D, Arnqvist G. Male-benefit sexually antagonistic genotypes show elevated vulnerability to inbreeding. BMC evol biol. 2017 Dec;17(1):134.
3. Hayman BI. The analysis of variance of diallel tables. Biometrics. 1954 Jun 1;10(2):235-44.
4. Griffing BR. Concept of general and specific combining ability in relation to diallel crossing systems. Aust j biol sci. 1956;9(4):463-93.
5. Cockerham CC, Weir BS. Quadratic analyses of reciprocal crosses. Biometrics. 1977 Mar 1:187-203.
6. Lenarcic AB, Svenson KL, Churchill GA, Valdar W. A general Bayesian approach to analyzing diallel crosses of inbred strains. Genetics. 2012 Feb 1;190(2):413-35.
7. Lynch M, Walsh B. Genetics and analysis of quantitative traits. Sunderland, MA: Sinauer; 1998.
8. VSN International. Genstat for Windows 18th Edition VSN International [software]. Hemel Hempstead, UK. 2015 Available from: [Genstatcouk](http://genstat.co.uk/).
9. Crowley JJ, Kim Y, Lenarcic AB, Quackenbush CR, Barrick CJ, Adkins DE, Shaw GS, Miller DR, de Villena FP, Sullivan PF, Valdar W. Genetics of adverse reactions to haloperidol in a mouse diallel: a drug-placebo experiment and Bayesian causal analysis. Genetics. 2013 Jan 1:genetics-113.
10. Payne RW. Procedure FDIALLEL In Genstat Release 18 Reference Manual, Part 3 Procedure Library PL26. [software]. Hemel Hempstead: VSN International. 2015.
11. Lenarcic AB. BayesDiallel: BayesDiallel Software R package version 098 [software]. 2014.
12. R Core Team R: A language and environment for statistical computing [software]. R foundation for statistical computation, Vienna, Austria. 2015. Available from: [http://wwwR-projectorg/](http://www.R-project.org/).
13. Kass RE, Raftery AE. Bayes factors. Journal of the american statistical association. 1995 Jun 1;90(430):773-95.
